# Supplementary material for: Altered chromatin topologies caused by balanced chromosomal translocation lead to central iris hypoplasia
Source: Nat Commun. 2024 Jun 13;15:5048. doi: 10.1038/s41467-024-49376-w (PMC11176186; doi:10.1038/s41467-024-49376-w)
Supplement: Supplementary file 1 — Supplementary information [file 41467_2024_49376_MOESM1_ESM.pdf]

# Altered chromatin topologies caused by balanced chromosomal translocation lead to central iris hypoplasia

Wenmin Sun[1#], Dan Xiong[2#], Jiamin Ouyang[1#], Xueshan Xiao[1#], Yi Jiang[1], Yingwei Wang[1], Shiqiang Li[1], Ziying Xie[2], Junwen Wang[1], Zhonghui Tang[2*], Qingjiong Zhang[1*]

[1] State Key Laboratory of Ophthalmology, Zhongshan Ophthalmic Center, Sun Yat-sen University, Guangdong Provincial Key Laboratory of Ophthalmology and Visual Science, Guangzhou 510060, China

[2] Zhongshan School of Medicine, Sun Yat-sen University, Guangzhou 510080, China

*Corresponding to:
Qingjiong Zhang, MD, PhD, zhangqji@mail.sysu.edu.cn
or
Zhonghui Tang PhD, tangzhh99@mail.sysu.edu.cn

[#]These authors contribute equally

Subject terms: noncoding structural variation; three-dimensional chromatin structure; central iris hypoplasia; APCDD1

**Supplementary Tables**

Supplementary Table 1. Clinical data of affected members from the family.

| ID | Age at Exam (yrs) | Gender | BCVA | Pupil size (mm) | IOP (mmHg) OD; OS | Corneal parameter (mm) | | ACD (mm) OD; OS | Lens | Axial length OD; OS (mm) |
|---|---|---|---|---|---|---|---|---|---|---|
| | | | | | | Diameter OD; OS | CCT OD; OS | | | |
| III:3 | 72 | F | 0.1* | 8 | / | / | / | / | Age-related cataract | 24.77; 24.13 |
| IV:4 | 55 | F | 0.3* | 8 | / | / | / | / | Age-related cataract | 25.32; 25.31 |
| IV:6 | 39 | F | 0.5 | 8 | 16.0; 14.9 | 12; 12 | 0.61; 0.61 | 2.76; 2.80 | / | 21.21; 21.55 |
| V:1 | 37 | M | 0.6 | 8 | / | / | / | / | / | 23.11; 23.14 |
| V:4 | 31 | M | 0.6 | 6 | 17; 15 | 11; 11 | 0.58; 0.60 | 2.63; 2.67 | / | 23.00; 22.87 |
| V:6 | 6 | F | 0.6 | 8 | 14.0; 16.0 | 12; 12 | 0.55; 0.55 | 2.55; 2.41 | / | 19.35; 19.17 |

Notes: BCVA, best corrected visual acuity. OD, right eye. OS, left eye. IOP, intraocular pressure. CCT, central corneal thickness. ACD, anterior chamber depth. *, the lower BCVAs in the two patients are due to partially lens opacity of age-related cataract. All the patients had the first symptom of photophobia with onset during first few months after birth.

Supplementary Table 2. Two-point LOD scores between central iris hypoplasia and markers at 6q15-q23.3 and 18.p11.31-q12.1.

| Markers | Position | | LOD score at θ = | | | | | | | Zmax | θmax |
|---|---|---|---|---|---|---|---|---|---|---|---|
| | cM# | Mb* | 0 | 0.01 | 0.05 | 0.1 | 0.2 | 0.3 | 0.4 | | |
| chr6 (q15-q23.3) | | | | | | | | | | | |
| D6S462 | 99.0 | 90.2 | -inf | -0.65 | -0.06 | 0.10 | 0.14 | 0.10 | 0.04 | 0.14 | 0.20 |
| D6S434 | 109.2 | 102.0 | 1.94 | 1.90 | 1.73 | 1.52 | 1.09 | 0.68 | 0.31 | 1.94 | 0 |
| D6S1592 | 113.6 | 105.9 | 1.94 | 1.9 | 1.73 | 1.52 | 1.09 | 0.68 | 0.31 | 1.94 | 0 |
| D6S1594 | 117.3 | 108.1 | 1.38 | 1.34 | 1.21 | 1.03 | 0.70 | 0.40 | 0.16 | 1.38 | 0 |
| D6S287 | 122.0 | 119.2 | 2.65 | 2.59 | 2.37 | 2.08 | 1.50 | 0.92 | 0.40 | 2.65 | 0 |
| D6S262 | 130.0 | 131.4 | 1.58 | 1.55 | 1.40 | 1.22 | 0.85 | 0.50 | 0.20 | 1.58 | 0 |
| D6S292 | 137.0 | 136.0 | -inf | -1.51 | -0.28 | 0.12 | 0.30 | 0.25 | 0.12 | 0.30 | 0 |
| chr18 (p11.31-q12.1) | | | | | | | | | | | |
| D18S452 | 18.7 | 5.8 | -inf | -3.70 | -1.70 | -0.93 | -0.30 | -0.06 | 0.02 | 0.02 | 0 |
| D18S1150 | 37.2 | 10.2 | 2.20 | 2.15 | 1.97 | 1.73 | 1.25 | 0.78 | 0.35 | 2.20 | 0 |
| D18S53 | 41.2 | 11.5 | 2.65 | 2.59 | 2.37 | 2.08 | 1.50 | 0.92 | 0.40 | 2.65 | 0 |
| D18S71 | 43.5 | 12.6 | 2.78 | 2.72 | 2.49 | 2.19 | 1.57 | 0.95 | 0.37 | 2.78 | 0 |
| D18S1107 | 51.2 | 24.5 | 2.58 | 2.52 | 2.31 | 2.03 | 1.46 | 0.90 | 0.37 | 2.58 | 0 |
| D18S478 | 52.9 | 27.6 | 2.54 | 2.48 | 2.26 | 1.97 | 1.39 | 0.82 | 0.32 | 2.54 | 0 |
| D18S56 | 56.7 | 30.7 | -inf | 0.73 | 1.22 | 1.25 | 1.01 | 0.65 | 0.28 | 1.25 | 0.1 |
| haplotype D6S434, D6S1592, D6S1594, D6S287, D6S262, D18S1150, D18S53, D18S71, D18S1107, D18S478 | | | | | | | | | | | |
| haplotype | / | / | 3.08 | 3.03 | 2.81 | 2.52 | 1.93 | 1.31 | 0.67 | 3.08 | 0 |

Notes: #Marshfield database; *Build 38

Supplementary Table 3. The resolution of the translocation identified in members of the family.

| Methodology | Individual ID | Resolution |
|---|---|---|
| Karyotype | IV:6 | 46,XX,t(6;18)(q22.2;p11.2) |
| FISH | IV:6 | ish t(6;18)(6p+,18p+;6q+,18q+) |
| WES | III:3, IV:4, V:4, V:6 | No |
| WGS | V:4, V:6 | / |
| Nanopore | V:4 | chr6:121619304 & chr18:10432739 |
| Sanger | III:3, IV:4, IV:6, V:1, V:4, V:6 | chr6:121619304 & chr18:10432739 |
| Hi-C | IV:6 | chr6:121619304 & chr18:10432739 |

Notes: FISH, fluorescent in situ hybridization. WES, whole-exome sequencing. WGS, whole-genome sequencing. /, The translocation was not identified by standard analysis of short-read WGS analysis until *post hoc* bioinformatic analysis according to the breakpoint from Nanopore platform.

Supplementary table 4. Primers used for Sanger validation and RT-qPCR.

| Name | Forward primers (5’-3’) | Reverse primers (5’-3’) |
|---|---|---|
| der(18) | AGACCTCACACTCTGGCTTT | TCTCGAGTTTCAGCGTGAGT |
| der(6) | GATGATGCCCAACCACACTG | GCTACTCCCAGGCTACAACT |
| chr18 | CCAAAGACGAAGCTGGCAAA | GGACTCAGCGCAATTTCCAT |
| chr6 | GTCTGAGTCTGAAGGCCACT | AAGACGGGTGGTTTTGGTTG |
| APCDD1-RT-qPCR | TCCTTCATCCAGACAGCAGG | TGATGAACTCTGGGCCTGAC |
| GAPDH-RT-qPCR | GGACTCATGACCACAGTCCATGCC | TCAGGGATGACCTTGCCCACAG |
| Enhancer-WT-1 | TTGAGAGTCTGAGTAACATGACAGTAG (F1) | CCCCATGTCCTCACATTCTGTAT (R1) |
| Enhancer-WT-2 | TCCAAGAGTTGGAACATTACATCAG (F2) | GTGAGTGTGGTACAAGAAAATAGACC (R2) |
| Enhancer-KO | TTGAGAGTCTGAGTAACATGACAGTAG (F1) | GTGAGTGTGGTACAAGAAAATAGACC (R2) |
| apcdd1l-RT-qPCR | TACCACCGAAACTAGAGGGC | AGAGTAGGTTGGGACTTGGC |
| zf-actin-RT-qPCR | ATGGATGATGAAATTGCCGCAC | ACCATCACCAGAGTCCATCACG |

Supplementary Table 5. Reagent and resource information for immunofluorescence staining.

| Reagent | Company name | Catalog numbers | RRID | Clonality | Clone numbers | Application | Dilution |
|---|---|---|---|---|---|---|---|
| **Primary antibody** | | | | | | | |
| Anti-APCDD1 | Bioss | Bs-1565R | AB_10855399 | Polyclonal | / | IF | 1:200 |
| APCDD1 Polyclonal Antibody | Thermo Fisher Scientific | PA5-98605 | AB_2813218 | Polyclonal | / | sWes | 1:200 |
| GAPDH (14C10) Rabbit mAb | Cell Signaling Technology | 2118S | AB_561053 | Monoclonal | 14C10 | sWes | 1:200 |
| Podoplanin Monoclonal Antibody | Thermo Fisher Scientific | 14-5381-82 | AB_1210505 | Monoclonal | eBio8.1.1 (8.1.1) | IF | 1:200 |
| Anti-Actin, α-Smooth Muscle - FITC antibody | Sigma-Aldrich | F3777 | AB_476977 | Monoclonal | 1A4 | IF | 1:500 |
| Mouse CDO Antibody | R&D systems | AF2429-SP | AB_2078889 | Polyclonal | / | IF | 1:200 |
| Anti-β-Tubulin III antibody | Sigma-Aldrich | T8578 | AB_1841228 | Monoclonal | 2G10 | IF | 1:500 |
| H3K27ac | Active motif | 39133 | / | Polyclonal | / | CUT&Tag | 1:50 |
| **Secondary antibody** | | | | | | | |
| Anti-Rabbit IgG (H+L) | Sigma-Aldrich | SAB3700894 | / | Polyclonal | / | CUT&Tag | 1:50 |
| Donkey Anti-Mouse IgG H&L (Alexa Fluor® 488) | Abcam | ab150105 | AB_2732856 | Polyclonal | / | IF | 1:1000 |
| Donkey Anti-Rabbit IgG H&L (Alexa Fluor® 568) | Abcam | ab175470 | AB_2783823 | Polyclonal | / | IF | 1:1000 |
| **Other reagents** | | | | | | | |
| DAPI | Siama-Aldrich | D9542 | / | / | / | IF | 1:5000 |
| TrueBlack® Lipofuscin Autofluorescence Quencher | Biotium | 23007 | / | / | / | IF | 1:20 |
| Actin-Tracker Green-488 (anti-phalloidin) | Beyotime | C2201S | / | / | / | IF | 1:100 |

Note: IF, immunofluorescence; sWes, Simple western analysis; /, unavailability.

## Supplementary figures

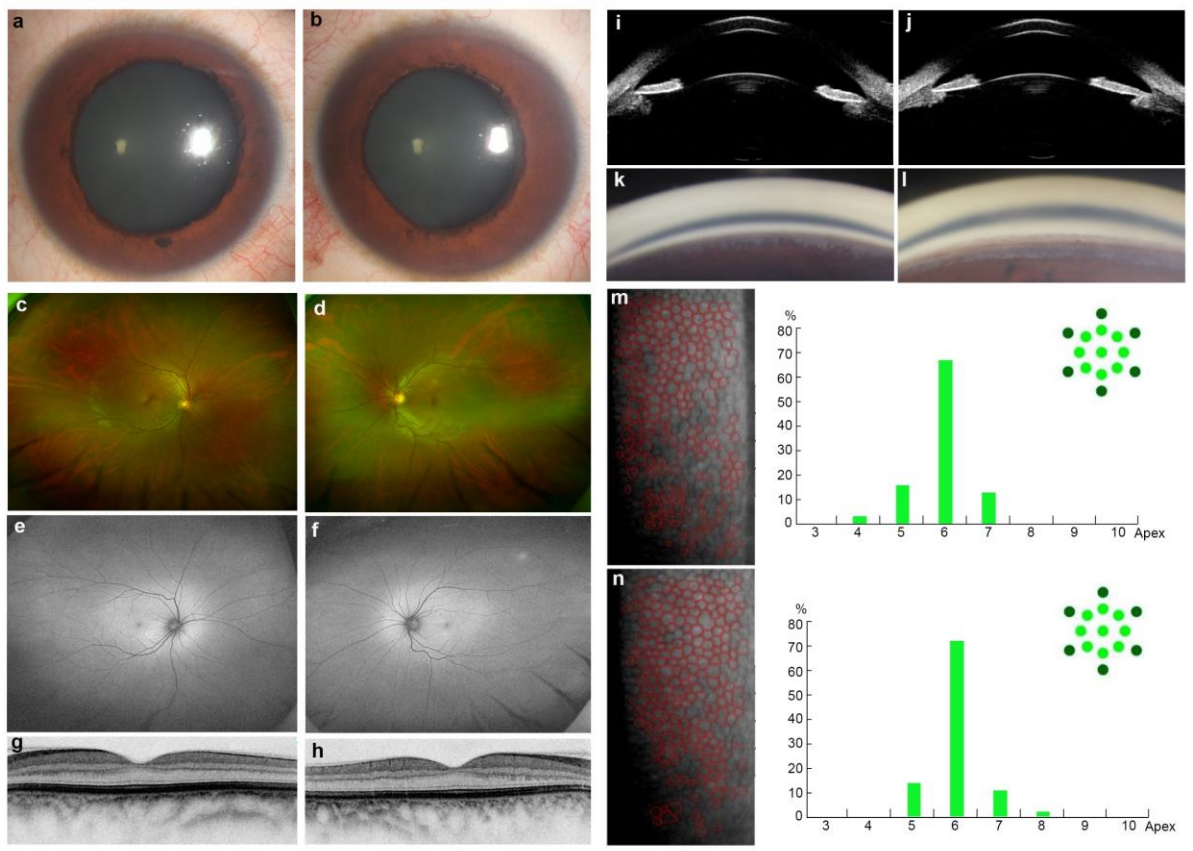


Supplementary Fig.1. Clinical features of the central iris hypoplasia. The patient with central iris hypoplasia showed an absence of the iris pupillary zone (a-b). The wide-field fundus and autofluorescence imaging showed a normal-like fundus (c-f) with normal foveal pit by OCT scan (g,h). Ultrasound biomicroscopy (UBM) showed the anterior chamber angle is open all around (I,j). Malformations of the anterior chamber angle were observed using the gonioscopy, including a relatively posterior attachment of iris and an increasing presence of pectinate ligament (k,l). The corneal endothelial cell density of the patient V:6 was 3003 cells/mm$^2$ with hexagonal cells of 68% in the right eye (m) and 2892 cells/mm$^2$ with hexagonal cells of 73% in the left eye (n).

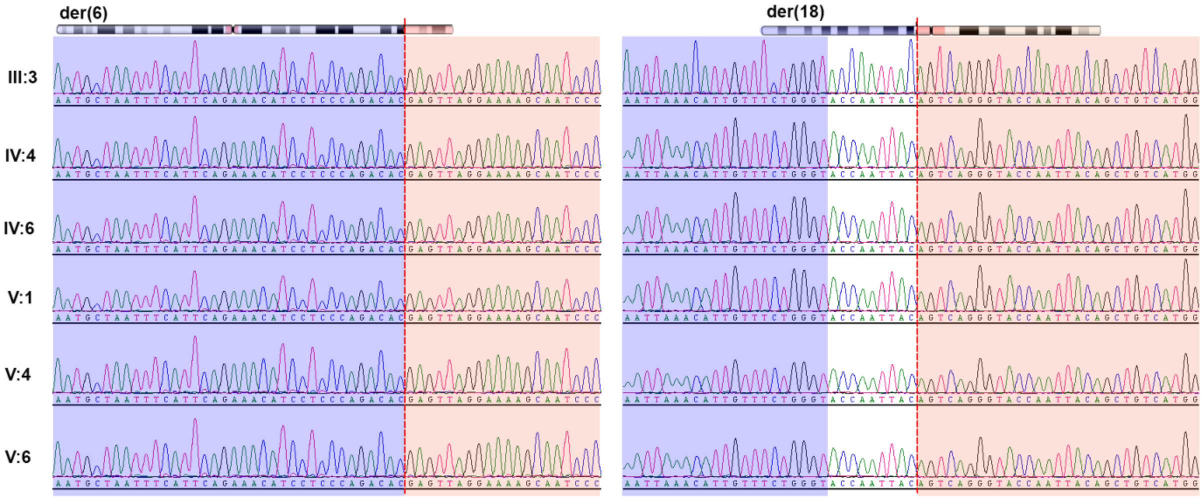


Supplementary Fig.2. Sequence chromatograms of two breakpoints of the translocation in the 6 affected members of the family. The left panel is structurally variant sequences flanking breakpoints of the translocation in the derived chromosome 6. The right panel is structurally variant sequences flanking breakpoints of the translocation in the derived chromosome 18.

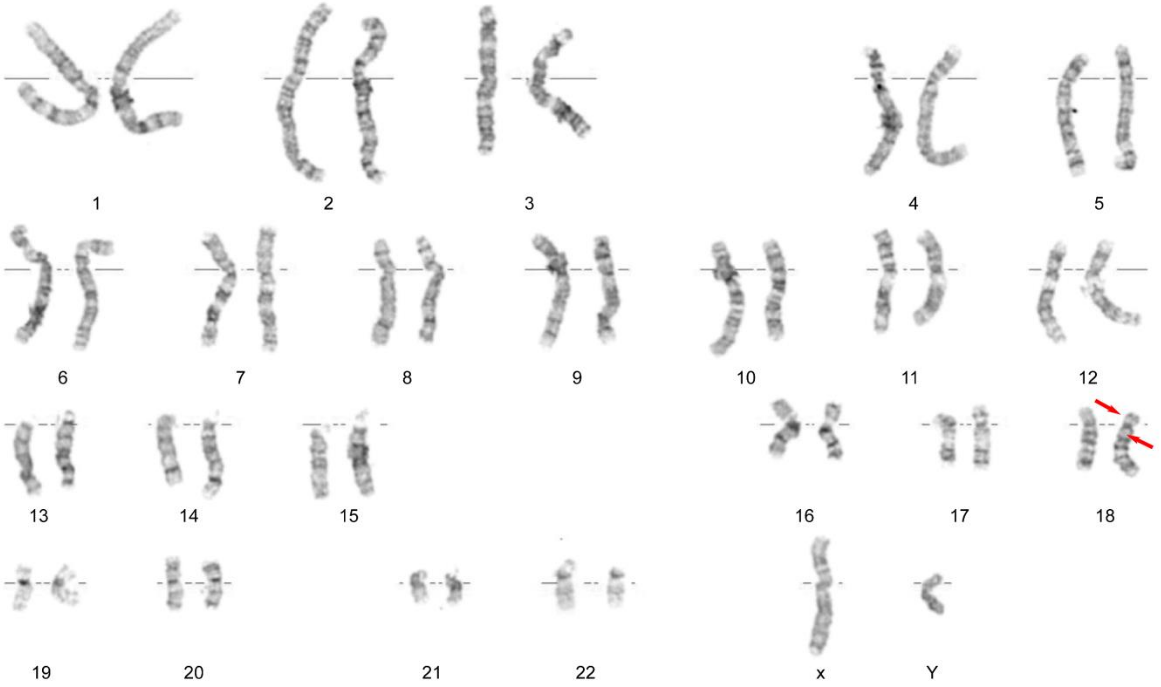


Supplementary Fig.3. Karyotype of the proband #713867.
A pericentric inversion of chromosome 18, inv(18)(p11.2 q11.2), was present in the proband #713867 (red arrows).

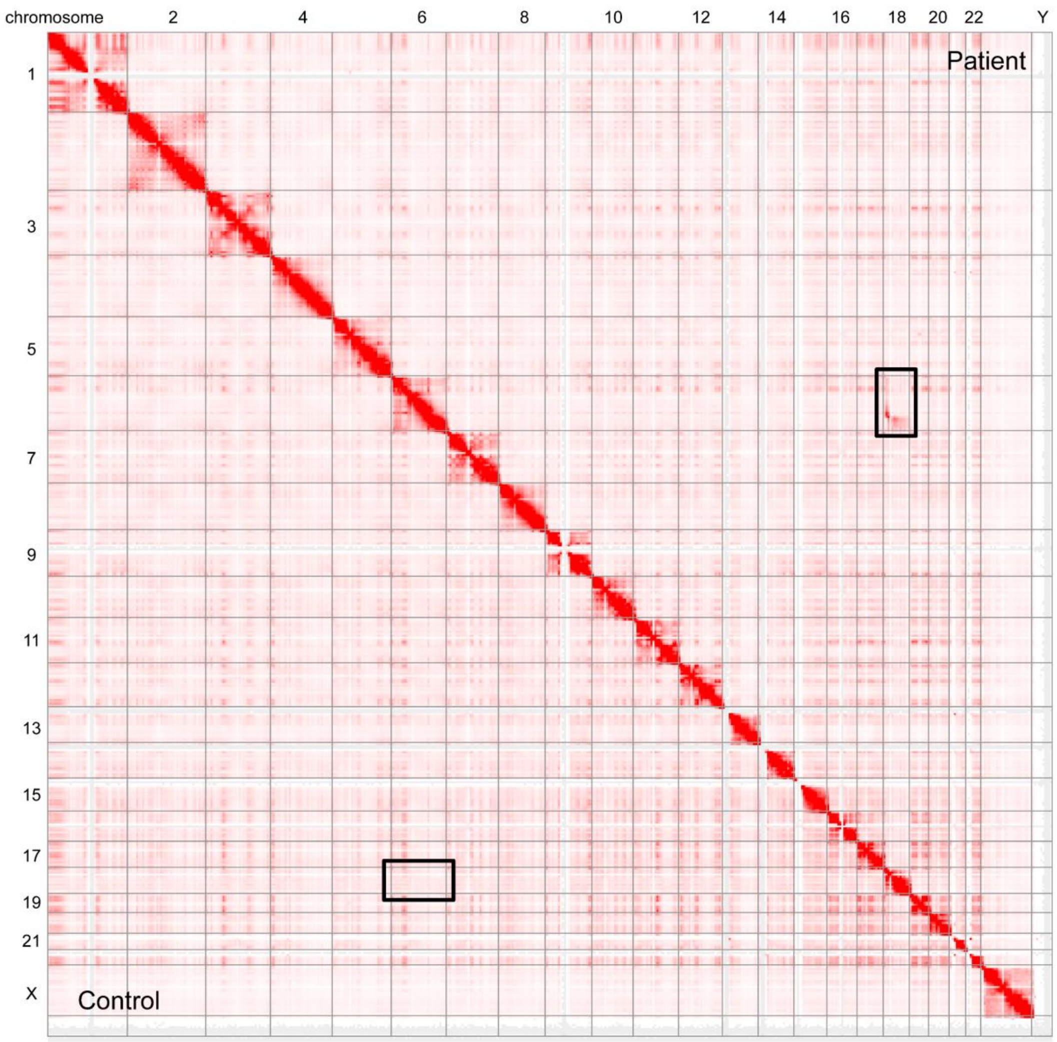


Supplementary Fig.4. Whole-genome Hi-C contact maps of iPSCs from the patient and the normal control.
The heatmap displays the interaction frequency between all possible genomic loci across the entire genome, binned at a resolution of 500-kb. The upper triangle represents the contact map from the patient sample, while the lower triangle represents the contact map from the normal control. The X and Y axes represent the genomic coordinates of each chromosome. Red colors indicate higher interaction frequency, while white colors indicate lower frequency. The black boxes highlight interactions between chromosome 6 and chromosome 18.

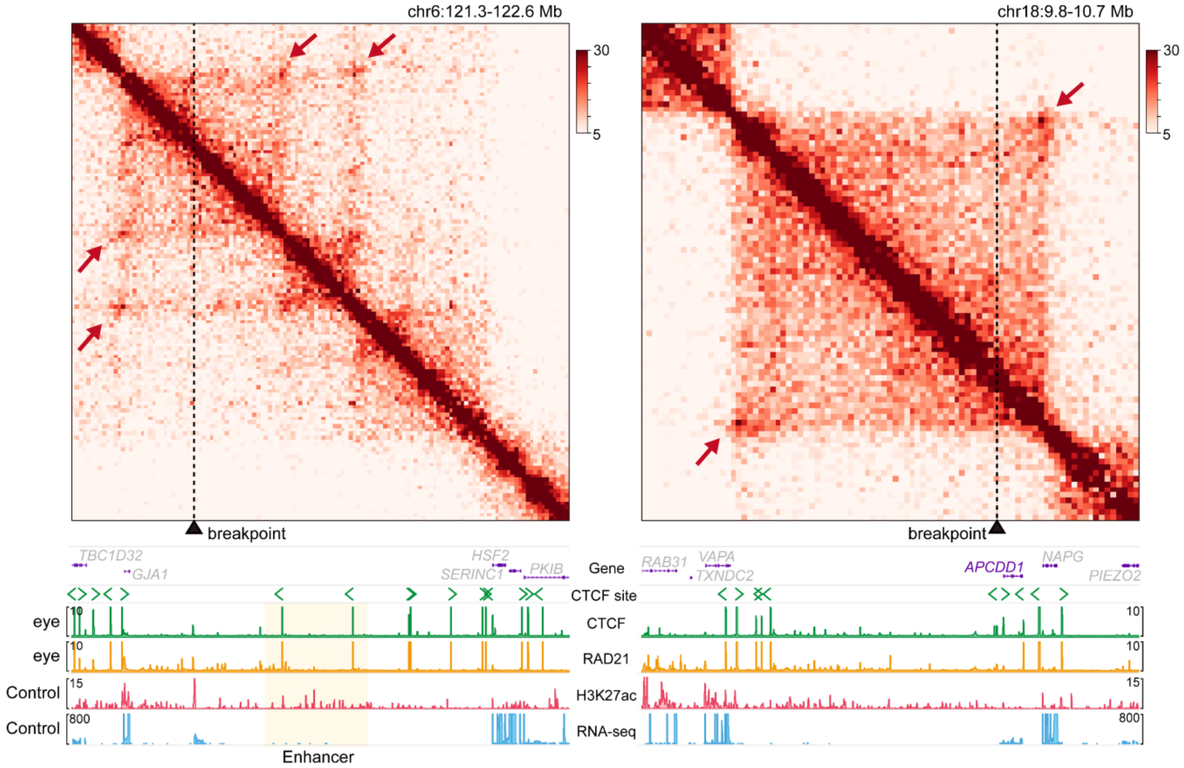


Supplementary Fig.5. Hi-C contact maps of iPSCs from the normal control around the breakpoints of the translocation.

The heatmap shows the interaction frequency between genomic regions surrounding the breakpoints of the translocation in iPSCs from the normal control, with binning at a resolution of 10-kb. The X and Y axes represent the genomic coordinates of each region. Red colors indicate higher interaction frequency, while white colors indicate lower frequency. The dash lines and black triangles indicate the locations of the two breakpoints. The bottom tracks were genes, CTCF site, CTCF peaks and RAD21 peaks from eye tissue, H3K27ac peaks and gene expression level of iPSC from the normal control, respectively. The orange box highlights an enhancer cluster, which exhibits strong activity in the iPSCs from the normal control. CTCF, CCCTC-Binding Factor.

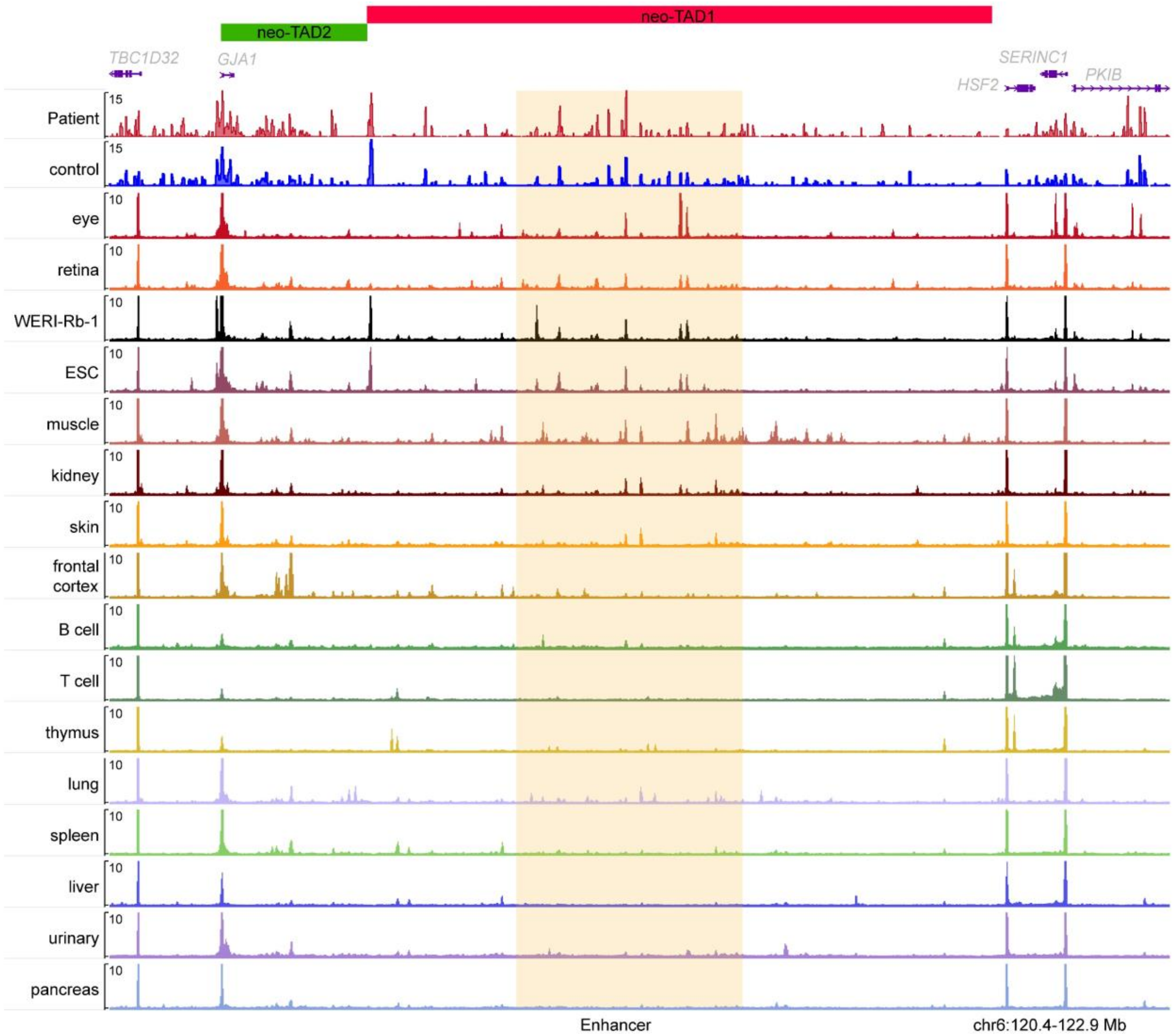


Supplementary Fig.6. H3K27ac activity of the enhancer cluster on chromosome 6 across 16 different tissues from Epimap project.

The tracks show the H3K27ac signal intensity of the enhancer cluster (highlighted in yellow) in various tissues. The enhancer cluster exhibits strong activities in the eye, retina, and ESC, while displaying weaker activity in other tissues. ESC, embryonic stem cell.

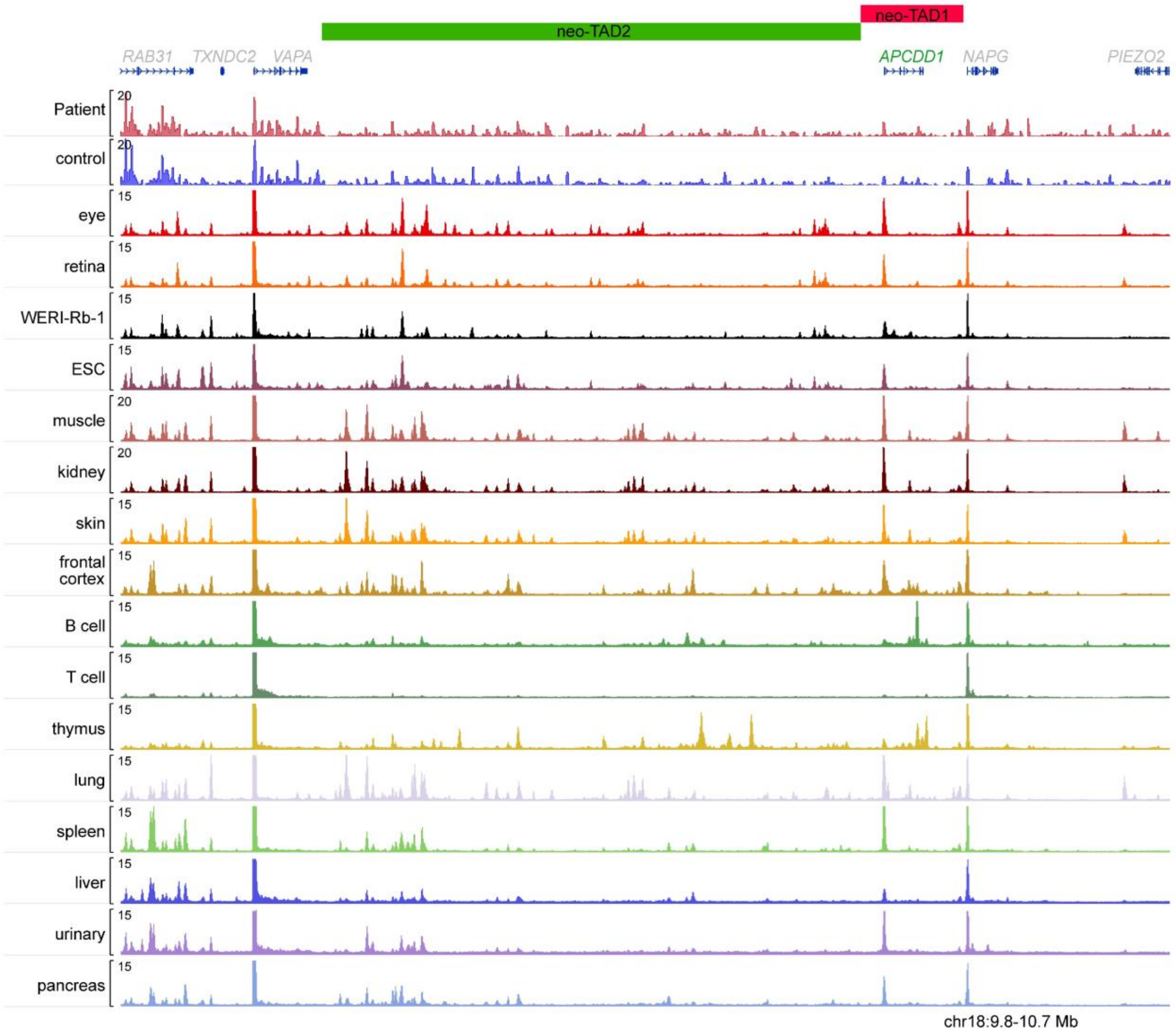


Supplementary Fig.7. H3K27ac activity of the enhancer cluster on chromosome 18 across 16 different tissues from Epimap project.

The enhancer cluster does not exhibit enrichment in specific tissues. ESC, embryonic stem cell.

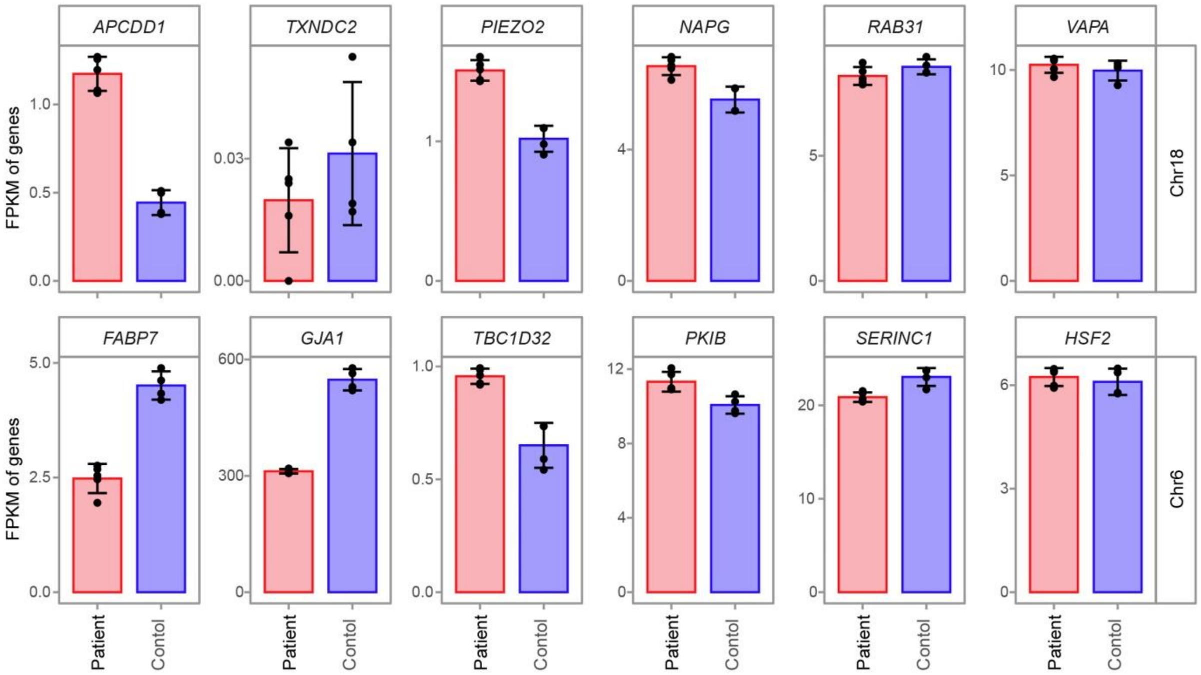


Supplementary Fig.8. The quantifications at individual replicate level of all the 12 genes close to the two breakpoints according to RNA-seq. Source data are provided as a Source Data file.

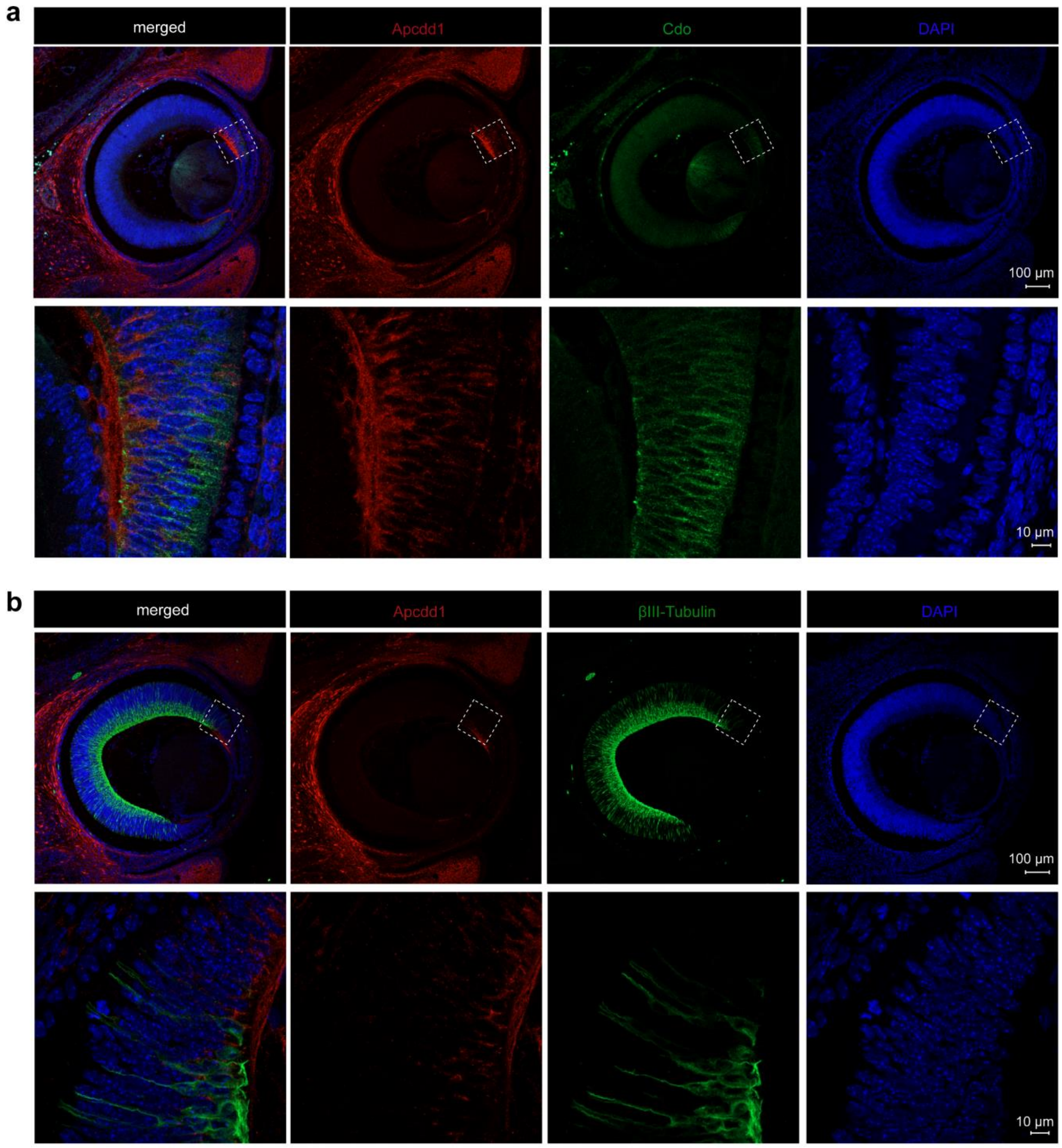


Supplementary Fig.9. Co-labelling with Apcdd1 and Cdo or βIII-Tubulin in embryonic mouse eyes at E14.5.

a, Apcdd1 staining is partly overlapped with Cdo of mouse eyes; b, Apcdd1 staining is separate from βIII-Tubulin of mouse eyes.

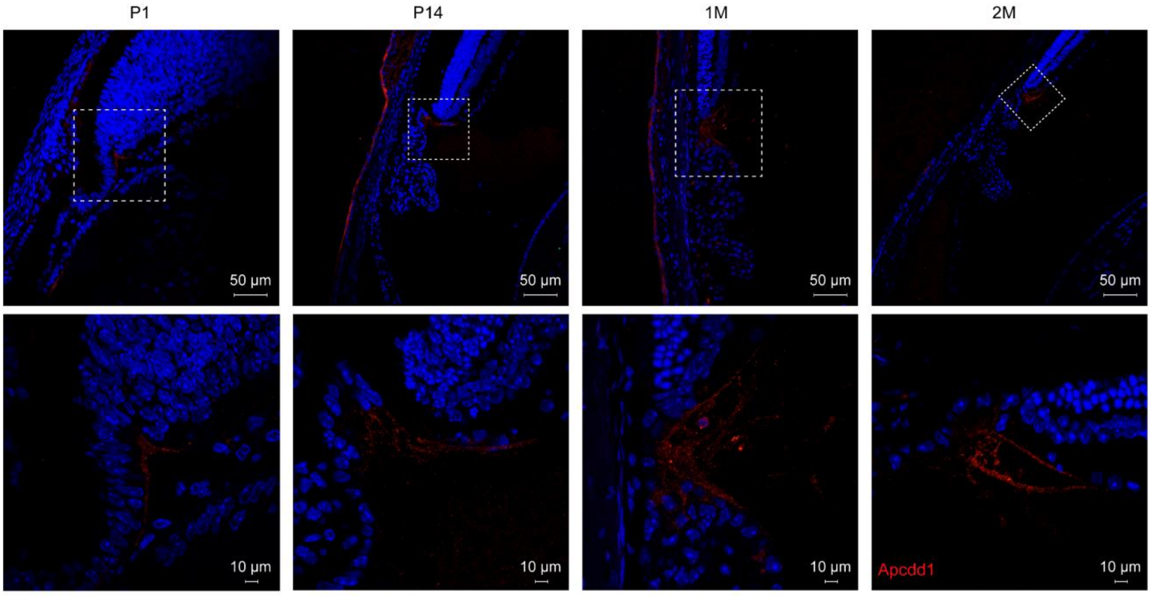


Supplementary Fig.10. Time-course of Apcdd1 expression in mouse eyes after birth.
Apcdd1 expression was confined to the boundary of ciliary body and retina in mouse eyes at different postnatal stages, namely P1, P14, 1M, 2M.

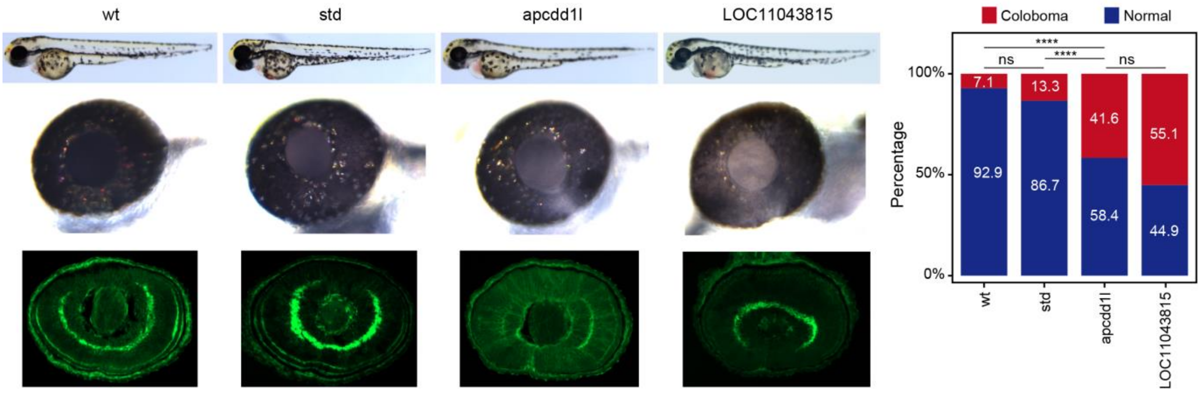


Supplementary Fig.11. Phenotypes of zebrafish larvae with overexpression of two paralogues, namely *apcdd1-like* (*apcdd1l*) and *LOC110438155*, of human *APCDD1*. Ocular coloboma is observed in *apcdd1l* overexpressed larvae and *LOC11043815* overexpressed larvae at day 3 post-fertilization (red arrowhead), which is confirmed by the optic fissure closure failure by immunofluorescence of phalloidin (red arrowhead). Coloboma is observed in 41.6% (n=173) of *apcdd1l*-overexpressed larvae and 55.1% (n=341) of *LOC11043815* overexpressed larvae, which is significantly higher than in wt larvae (7.1%, n=155) and in std larvae (13.3%, n=113). Pearson's Chi-squared test was employed to assess the significance. ****, $P < 0.0001$. ns, no significance.
