# Supplementary material for: Altered chromatin topologies caused by balanced chromosomal translocation lead to central iris hypoplasia
Source: Nat Commun. 2024 Jun 13;15:5048. doi: 10.1038/s41467-024-49376-w (PMC11176186; doi:10.1038/s41467-024-49376-w)
Supplement: Supplementary file 3 — Description of Additional Supplementary Files [file 41467_2024_49376_MOESM3_ESM.pdf]

Description of additional supplementary files

Supplementary data legend:

File name: Supplementary data 1

Description: The quantifications at individual replicate level of all the 40 genes within 2.5 Mb of each breakpoint.

File name: Supplementary data 2

Description: The full data set of differential expression from RNA-seq.

File name: Supplementary data 3

Description: The whole report file of the Simple Western analysis for evaluation of APCDD1 protein in iPSCs from the patient (Lanes 10-13 in quadruplicate) and the control (Lanes 14-17 in quadruplicate), in which uncropped images for Fig. 3f (Lane 11 and Lane 16) is on page 6 and the correction quantities of APCDD1 (Fig.3g) are in page 12-13.
