# Supplementary material for: Altered chromatin topologies caused by balanced chromosomal translocation lead to central iris hypoplasia
Source: Nat Commun. 2024 Jun 13;15:5048. doi: 10.1038/s41467-024-49376-w (PMC11176186; doi:10.1038/s41467-024-49376-w)

File: 2022-12-07_13-12-39_Wes Size

Run

	2022-12-07_13-12-39_Wes Size
	F:\Project\2023 sub\QT342\01 SunW-APCDD1-NC rev8
	Wes Size
	Regular: 12-230 kDa
	Wes : Wes WS3312 - WS3312
	2.5.30766
	9573802022
	Wed 12:58 PM Dec 7, 2022 CST
	Wed 3:33 PM Dec 7, 2022 CST
	None

File: 2022-12-07_13-12-39_Wes Size

Protocol

Separation Matrix	
Well Row	
Load Time (sec)	
Stacking Matrix	
Well Row	
Load Time (sec)	
Sample	
Well Row	
Load Time (sec)	
Separation Time (min)	
Separation Voltage (volts)	
Standards Exposure (sec)	
EE Immobilization Time (sec)	
Antibody Diluent Time (min)	
Well Row	
Primary Antibody Time (min)	
Well Row	
Secondary Antibody Time (min)	
Well Row	
Detection	
Well Row	
Detection Profile	
Exposure 1 (sec)	
Exposure 2 (sec)	
Exposure 3 (sec)	
Exposure 4 (sec)	
Exposure 5 (sec)	
Exposure 6 (sec)	
Exposure 7 (sec)	
Exposure 8 (sec)	
Exposure 9 (sec)	

File: 2022-12-07_13-12-39_Wes Size

Plate Layout

<table>
<tr><th></th><th>1</th><th>2</th><th>3</th><th>4</th><th>5</th><th>6</th><th>7</th><th>8</th><th>9</th><th>10</th><th>11</th><th>12</th><th>13</th><th>14</th><th>15</th><th>16</th><th>17</th><th>18</th><th>19</th><th>20</th><th>21</th><th>22</th><th>23</th><th>24</th><th>25</th></tr>
<tr><td>A</td><td>Biot...</td><td>Cell...</td><td>Cell...</td><td>Cell...</td><td>Cell...</td><td>Cell...</td><td>Cell...</td><td>Cell...</td><td>Cell...</td><td>Pati...</td><td>Pati...</td><td>Pati...</td><td>Pati...</td><td>Cont...</td><td>Cont...</td><td>Cont...</td><td>Cont...</td><td>IPS1</td><td>IPS2</td><td>IPS3</td><td>IPS4</td><td>IPS1-2</td><td>IPS2-2</td><td>IPS3-2</td><td>IPS4-2</td></tr>
<tr><td>B</td><td colspan="25">Antibody Diluent</td></tr>
<tr><td>C</td><td>Bloc...</td><td colspan="24">AP(Thermo)1:200+gapdh1:200</td></tr>
<tr><td>D</td><td>Stre...</td><td colspan="24">Secondary HRP Conjugate</td></tr>
<tr><td>E</td><td colspan="25">Luminol/Peroxide</td></tr>
</table>

File: 2022-12-07_13-12-39_Wes Size

Analysis Settings

Hidden Capillaries	None
Images	
Luminescence (sec)	8.0
Lane Contrast	Slider
White Level	1117.7
Black Level	180337.1
Peak Fit	fit
Apply To	Default
Range Min (MW (kDa))	1.0
Range Max (MW (kDa))	250.0
Range View	Analysis
Baseline Threshold	1.0
Baseline Window (pixels)	15.0
Baseline Stiffness	1.0
Peak Find Threshold	10.0
Peak Find Width (pixels)	9.0
Peak Find Area Calculation	Gaussian Fit
Peak Name	Peak Group 1
Apply To	All
GAPDH (MW (kDa))	37
GAPDH Range (%)	20
AP (MW (kDa))	70
AP Range (%)	20
Loading Control	Loading Control 1
Peak Name Group	Peak Group 1
Peak Name	GAPDH
Control	Area: 10000.0
Ladder Settings	Cap: 1
Apply To	Default
Ladder Peak 1 (MW (kDa))	12.0
Ladder Peak 2 (MW (kDa))	40.0
Ladder Peak 3 (MW (kDa))	66.0
Ladder Peak 4 (MW (kDa))	116.0
Ladder Peak 5 (MW (kDa))	180.0
Ladder Peak 6 (MW (kDa))	230.0
Signal To Noise	
Algorithm	S/N Compass v5.0
Standards	Biotinylated Ladder (12kDa-230kDa)
Apply To	Default
Standard 1 (MW (kDa))	1
Standard 1 Position (pixels)	260
Standard 1 Fit	no
Standard 1 Registration	yes
Standard 2 (MW (kDa))	29
Standard 2 Position (pixels)	400
Standard 2 Fit	yes
Standard 2 Registration	no
Standard 3 (MW (kDa))	230

File: 2022-12-07_13-12-39_Wes Size

Standard 3 Position (pixels)	650
Standard 3 Fit	yes
Standard 3 Registration	no
Advanced	Advanced
Apply To	Default
Standards Peak Width (pixels)	15
Standards Allowable Drift (pixels)	100
Sample Peak Fit Starting Width Ratio	0.5
Image Median Filter Threshold Ratio	0.1
Image Median Filter Threshold Limit (counts)	10

File: 2022-12-07_13-12-39_Wes Size

Lanes

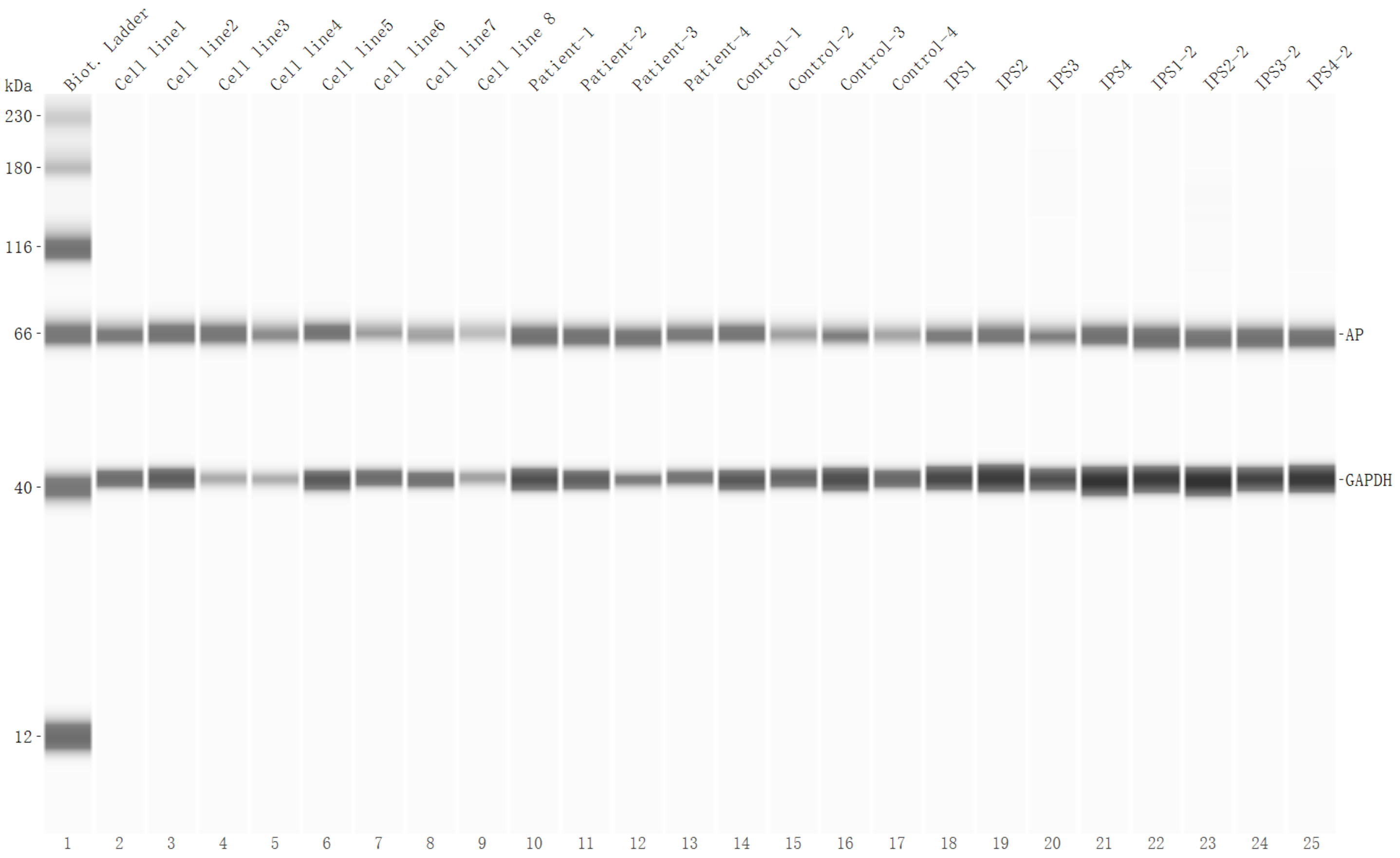

File: 2022-12-07_13-12-39_Wes Size

Standard

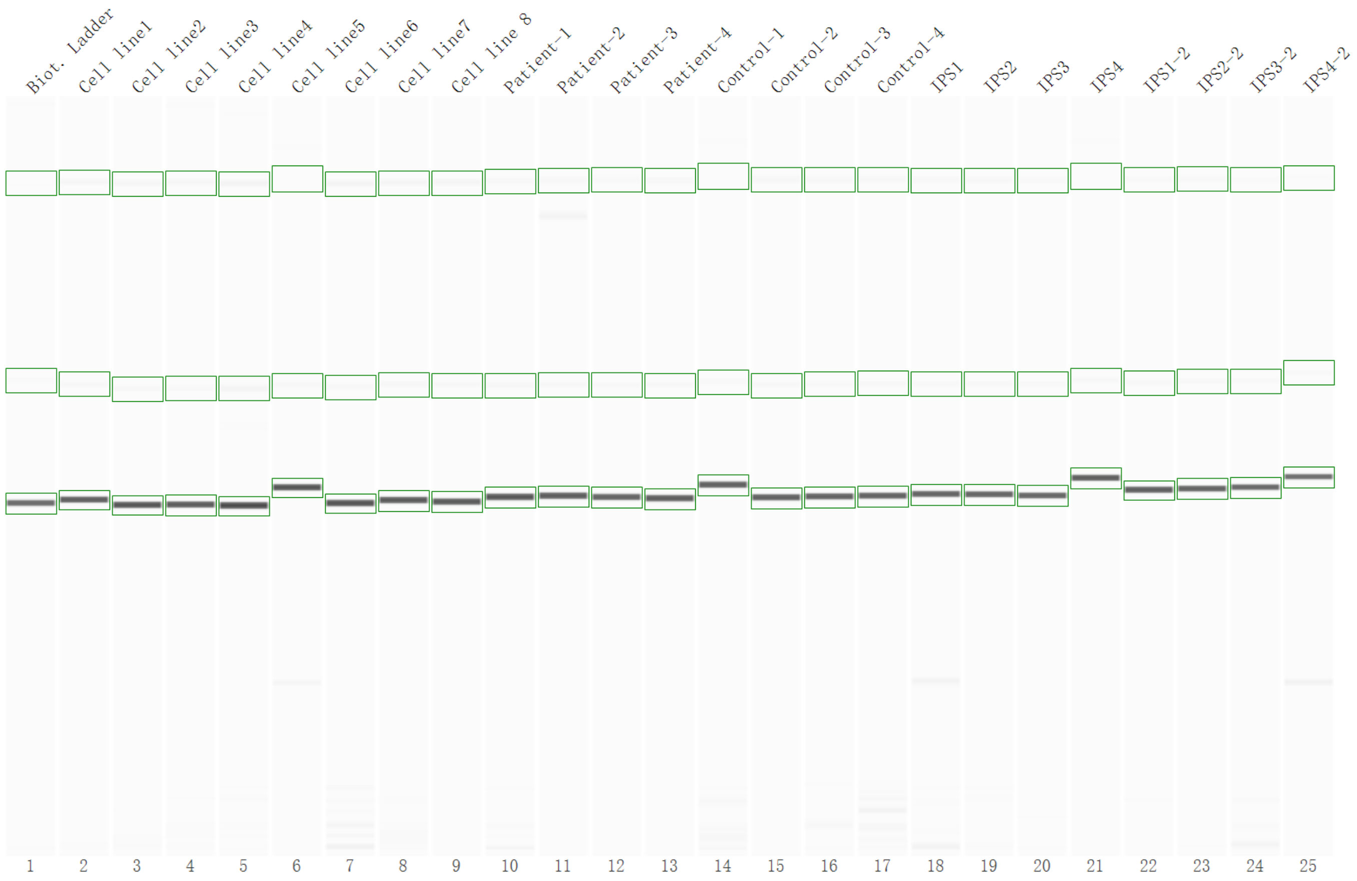

File: 2022-12-07_13-12-39_Wes Size

IV Plot

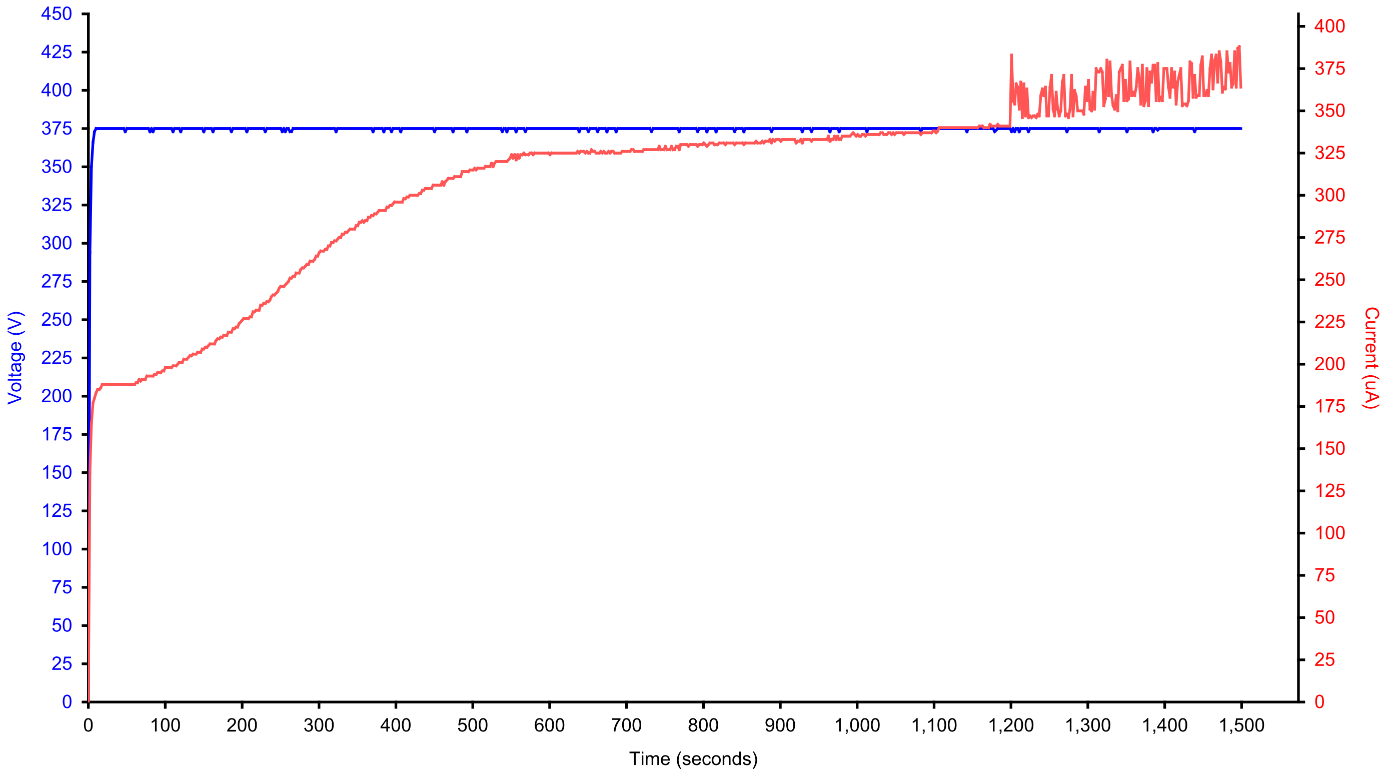

F:\Project\2023 sub\QT342\01 SunW-APCDD1-NC rev8\2022-12-07_13-12-39_Wes Size.cbz

File: 2022-12-07_13-12-39_Wes Size

Sample Plots

Biot. Ladder, Cap:1

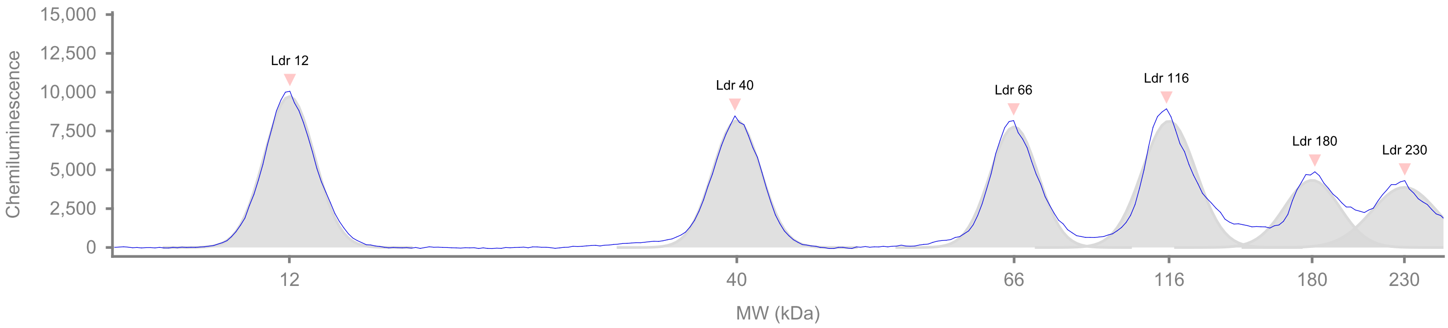

Cell line1, Cap:2

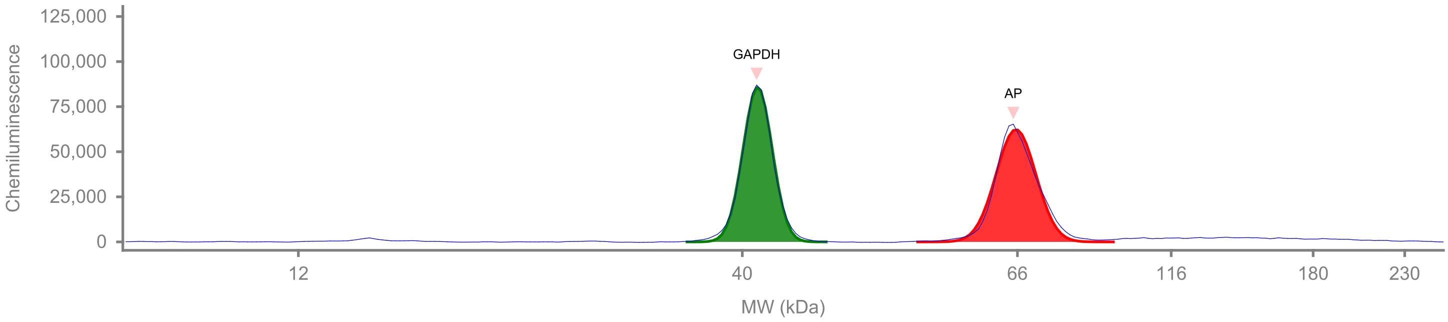

Cell line2, Cap:3

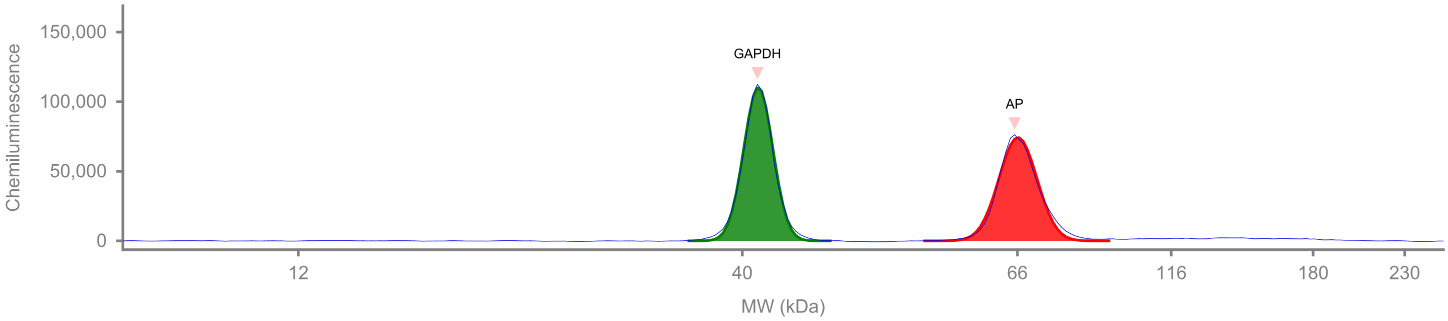

Cell line3, Cap:4

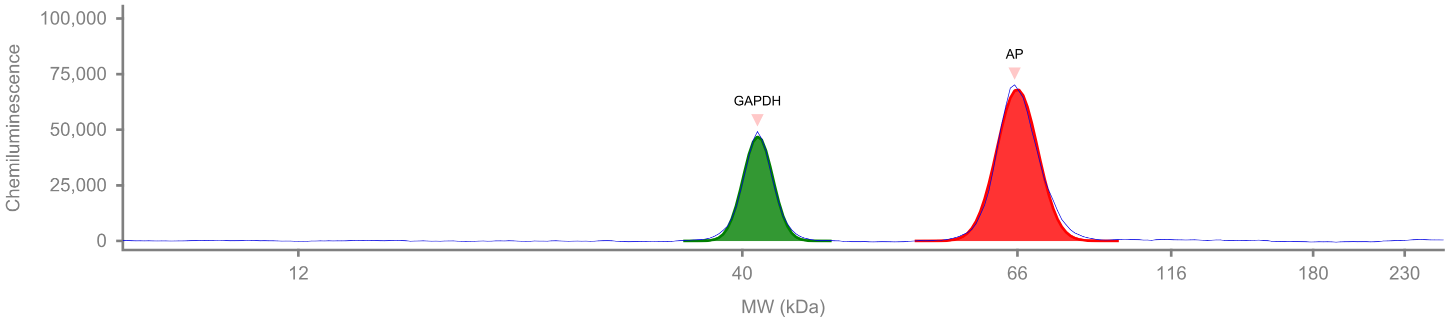

File: 2022-12-07_13-12-39_Wes Size

Cell line4, Cap:5

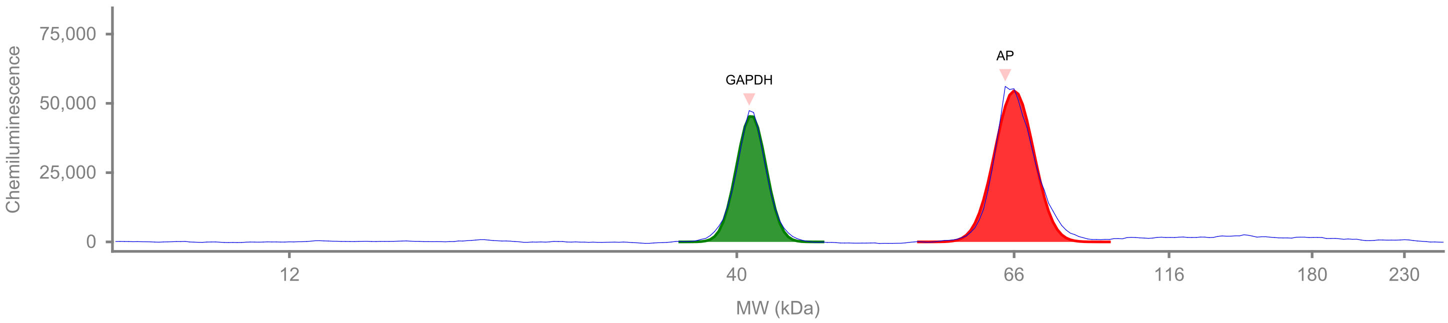

Cell line5, Cap:6

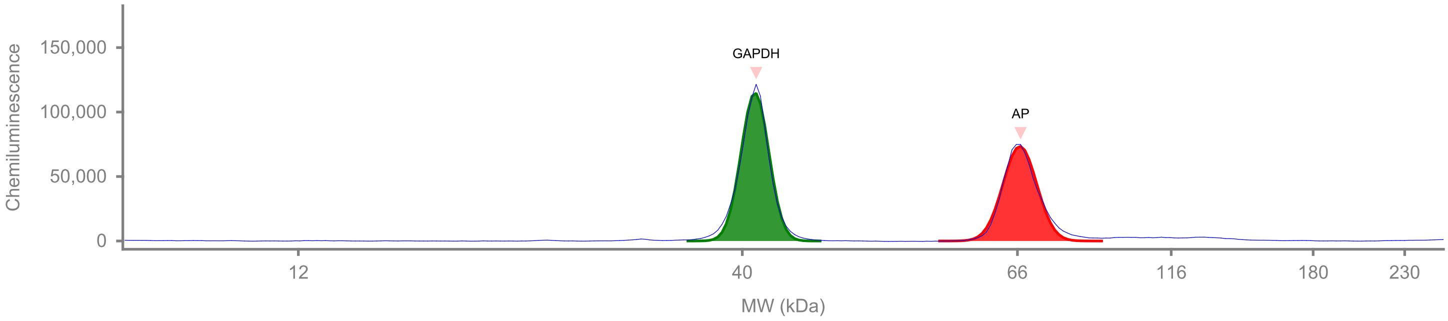

Cell line6, Cap:7

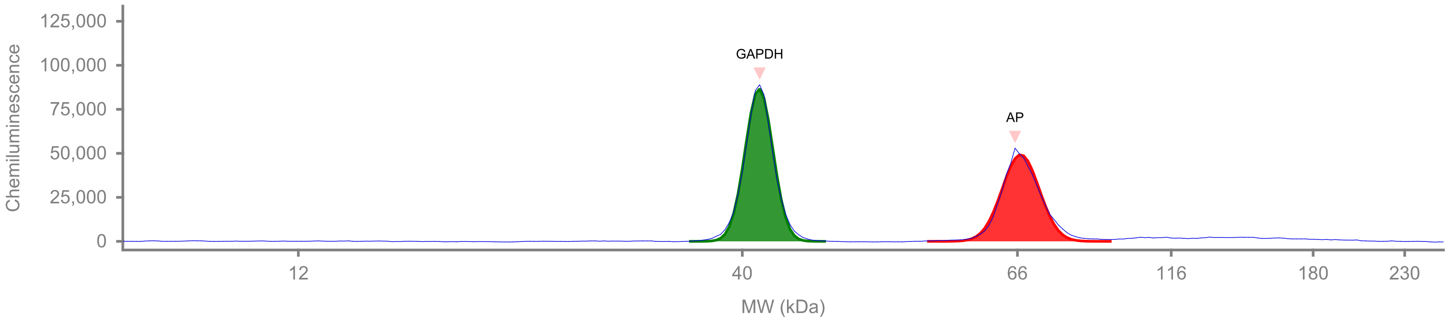

Cell line7, Cap:8

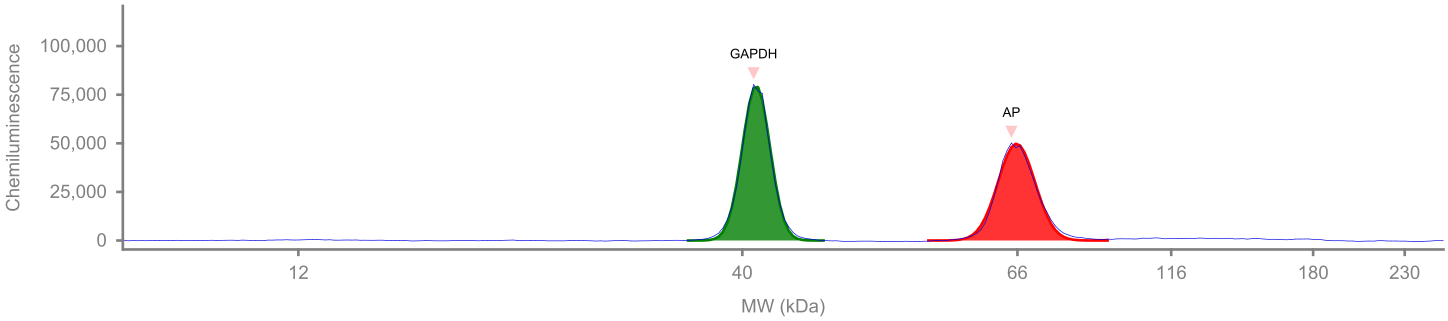

proteinsimple a biotechne brand

File: 2022-12-07_13-12-39_Wes Size

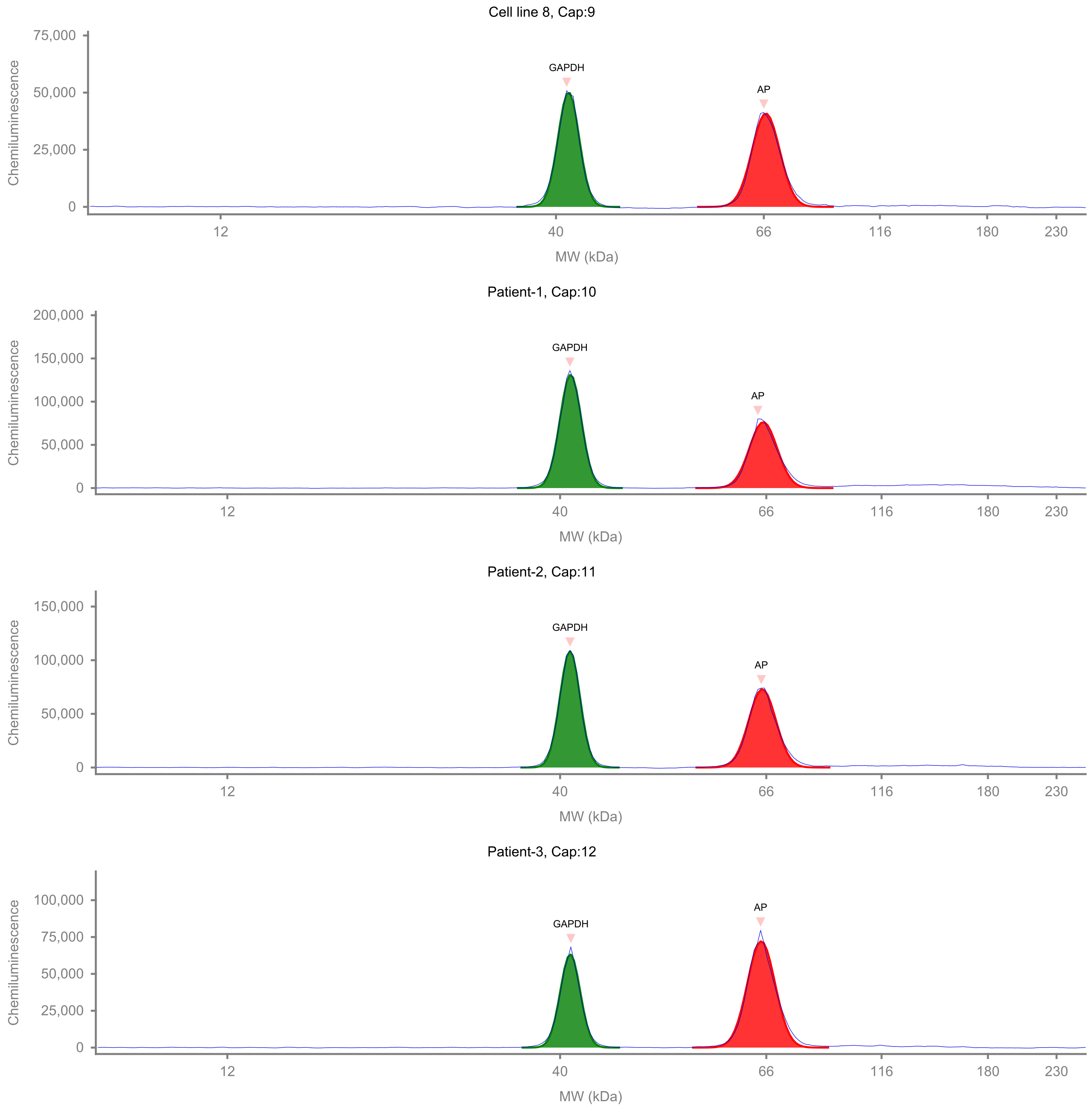

F:\Project\2023 sub\QT342\01 SunW-APCDD1-NC rev8\2022-12-07_13-12-39_Wes Size.cbz

File: 2022-12-07_13-12-39_Wes Size

Patient-4, Cap:13

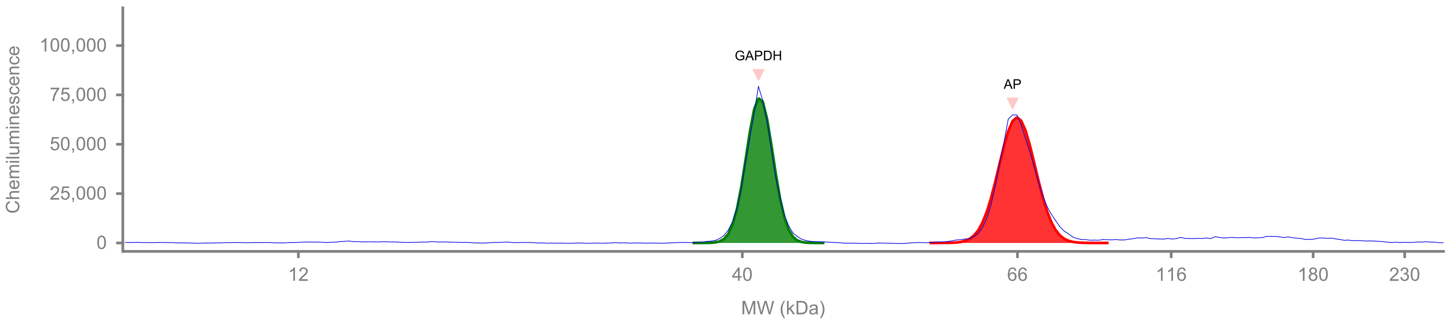

Control-1, Cap:14

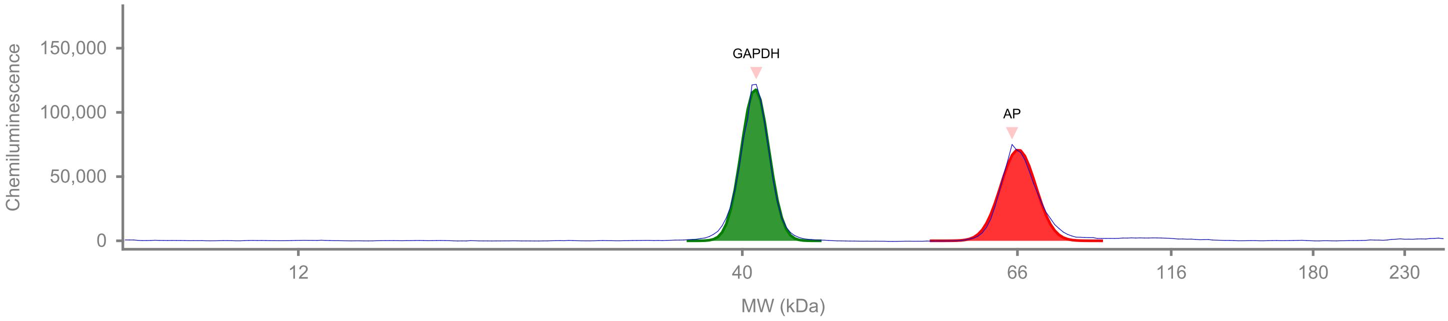

Control-2, Cap:15

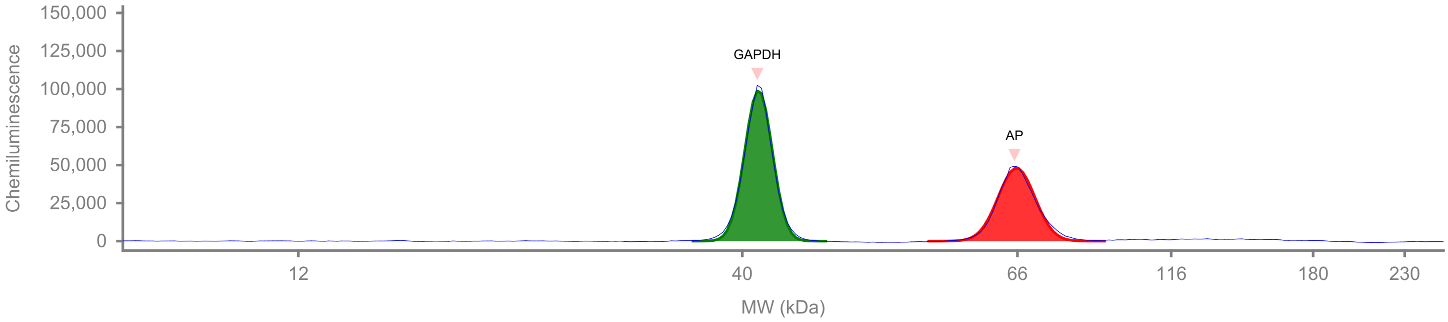

Control-3, Cap:16

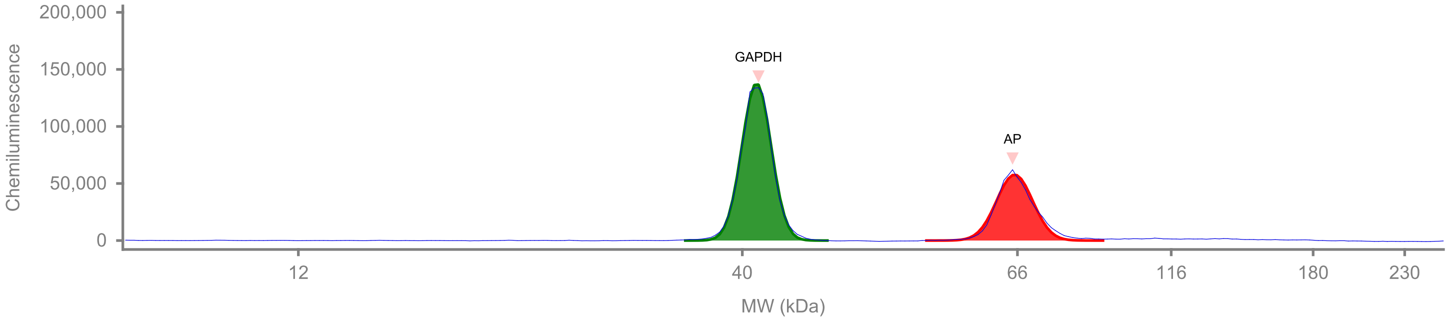

proteinsimple a biotechne brand

File: 2022-12-07_13-12-39_Wes Size

Control-4, Cap:17

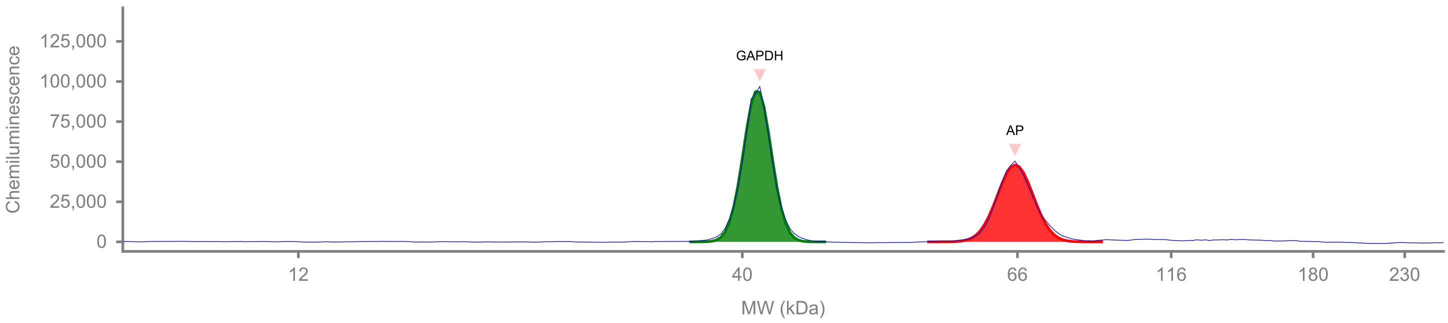

IPS1, Cap:18

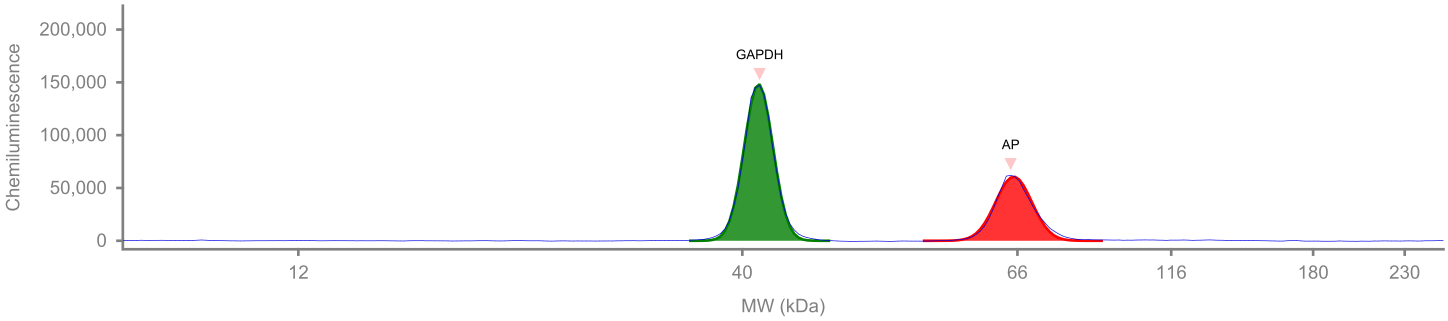

IPS2, Cap:19

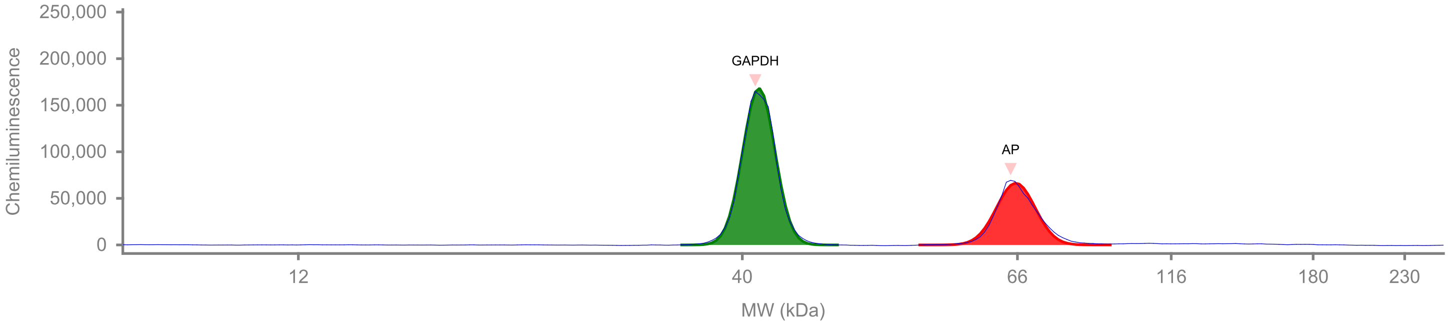

IPS3, Cap:20

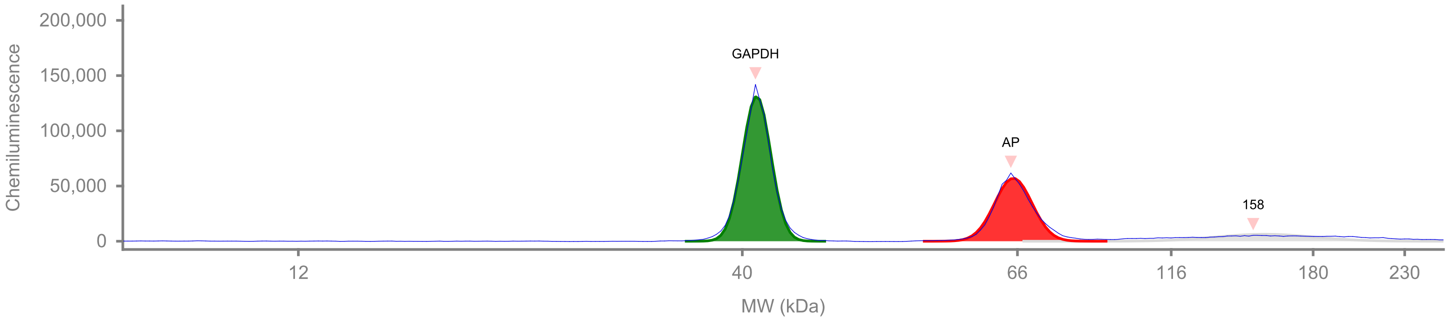

proteinsimple a biotechne brand

File: 2022-12-07_13-12-39_Wes Size

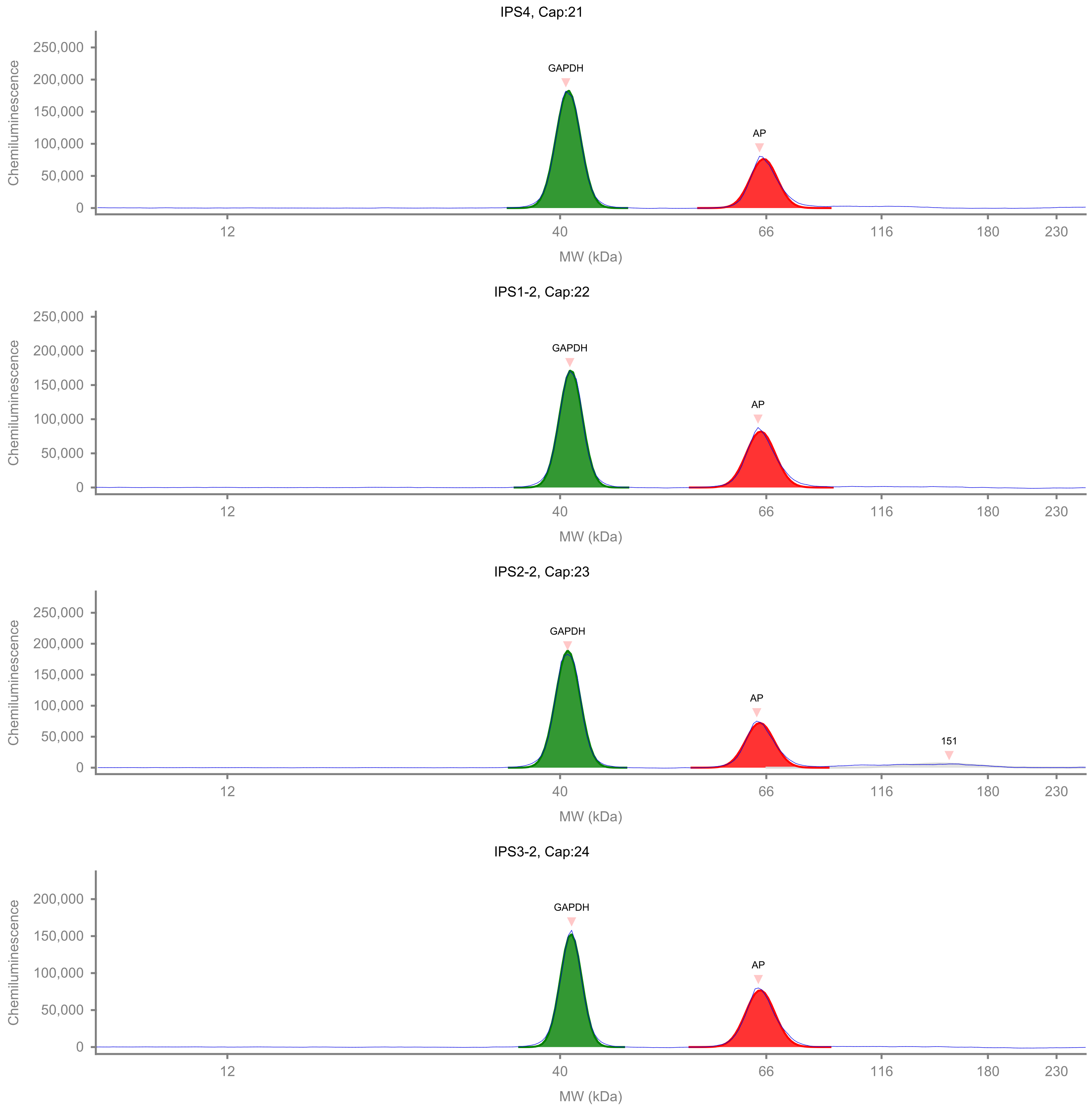

File: 2022-12-07_13-12-39_Wes Size

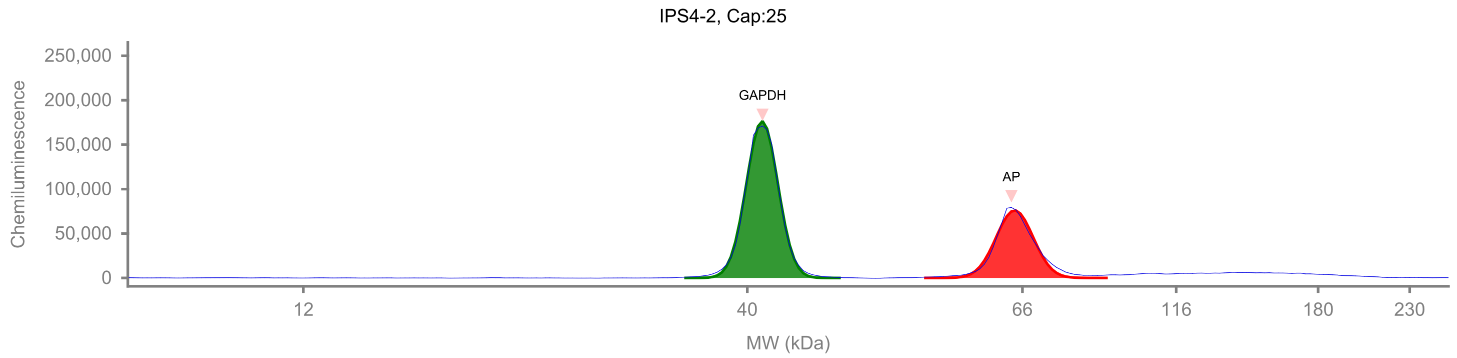

Sample Peaks

Biot. Ladder	Blocking	1	1	Ldr 12	434	12	9726.6	136394.1			13.2	304.0	51.4
Biot. Ladder	Blocking	1	2	Ldr 40	534	40	8166.4	109532.3			12.6	267.4	100.3
Biot. Ladder	Blocking	1	3	Ldr 66	595	66	7770.2	104329.2			12.6	263.5	150.1
Biot. Ladder	Blocking	1	4	Ldr 116	629	116	8135.7	122423.5			14.1	275.1	174.8
Biot. Ladder	Blocking	1	5	Ldr 180	662	180	4326.7	65956.5			14.3	125.4	196.8
Biot. Ladder	Blocking	1	6	Ldr 230	682	230	3872.7	70469.8			17.1	88.2	209.4
Cell line1	AP(Thermo)1:200+gapdh1:200	2	1	GAPDH	534	41	85995.1	718107.7	50.3	10000.0	7.8	1121.4	1193.2
Cell line1	AP(Thermo)1:200+gapdh1:200	2	2	AP	594	66	62371.0	710729.4	49.7	9897.3	10.7	761.4	1487.6
Cell line2	AP(Thermo)1:200+gapdh1:200	3	1	GAPDH	530	42	110397.1	934328.8	53.2	10000.0	8.0	1502.5	1463.6
Cell line2	AP(Thermo)1:200+gapdh1:200	3	2	AP	591	66	74365.4	822306.5	46.8	8801.0	10.4	966.2	1856.2
Cell line3	AP(Thermo)1:200+gapdh1:200	4	1	GAPDH	531	41	46944.5	407658.3	33.4	10000.0	8.2	645.4	1246.8
Cell line3	AP(Thermo)1:200+gapdh1:200	4	2	AP	591	66	68168.0	813779.0	66.6	19962.3	11.2	861.9	1579.2
Cell line4	AP(Thermo)1:200+gapdh1:200	5	1	GAPDH	530	41	45733.7	385264.5	38.9	10000.0	7.9	547.9	1407.8

File: 2022-12-07_13-12-39_Wes Size

Cell line4	AP(Thermo)1:200 +gapdh1:200	5	2	AP	591	66	54565.4	606064.2	61.1	15731.1	10.4	636.5	1662.7
Cell line5	AP(Thermo)1:200 +gapdh1:200	6	1	GAPDH	534	41	115011.8	933655.0	56.3	10000.0	7.6	1409.7	934.2
Cell line5	AP(Thermo)1:200 +gapdh1:200	6	2	AP	596	67	73468.6	725243.4	43.7	7767.8	9.3	912.2	960.8
Cell line6	AP(Thermo)1:200 +gapdh1:200	7	1	GAPDH	532	42	86708.7	691535.4	56.7	10000.0	7.5	1259.0	1238.2
Cell line6	AP(Thermo)1:200 +gapdh1:200	7	2	AP	592	67	49260.8	527941.9	43.3	7634.3	10.1	695.7	1392.0
Cell line7	AP(Thermo)1:200 +gapdh1:200	8	1	GAPDH	534	41	79720.1	632504.2	54.6	10000.0	7.5	1107.8	1353.6
Cell line7	AP(Thermo)1:200 +gapdh1:200	8	2	AP	593	66	49816.5	526058.2	45.4	8317.1	9.9	687.4	1723.9
Cell line 8	AP(Thermo)1:200 +gapdh1:200	9	1	GAPDH	534	42	50292.9	405448.5	48.3	10000.0	7.6	658.3	1338.7
Cell line 8	AP(Thermo)1:200 +gapdh1:200	9	2	AP	594	67	40913.6	433704.5	51.7	10696.9	10.0	526.8	1850.1

File: 2022-12-07_13-12-39_Wes Size

Patient-1	AP(Thermo)1:200+gapdh1:200	10	1	GAPDH	533	41	131410.1	1090377.1	57.0	10000.0	7.8	1588.6	1337.2
Patient-1	AP(Thermo)1:200+gapdh1:200	10	2	AP	593	66	76361.9	821907.2	43.0	7537.8	10.1	876.7	1520.8
Patient-2	AP(Thermo)1:200+gapdh1:200	11	1	GAPDH	534	41	108619.2	846551.3	52.4	10000.0	7.3	1674.2	1348.5
Patient-2	AP(Thermo)1:200+gapdh1:200	11	2	AP	593	65	72986.3	767851.7	47.6	9070.3	9.9	1082.8	1534.2
Patient-3	AP(Thermo)1:200+gapdh1:200	12	1	GAPDH	534	41	63392.9	491207.1	38.7	10000.0	7.3	1004.5	1103.8
Patient-3	AP(Thermo)1:200+gapdh1:200	12	2	AP	593	65	72209.3	779216.4	61.3	15863.3	10.1	1104.8	1130.4
Patient-4	AP(Thermo)1:200+gapdh1:200	13	1	GAPDH	534	42	73594.5	568345.4	45.9	10000.0	7.3	1066.1	1156.5
Patient-4	AP(Thermo)1:200+gapdh1:200	13	2	AP	594	66	63624.7	670406.0	54.1	11795.7	9.9	907.7	1383.5
Control-1	AP(Thermo)1:200+gapdh1:200	14	1	GAPDH	537	41	118257.6	955106.6	56.7	10000.0	7.6	1268.4	957.0

proteinsimple a biotechne brand

File: 2022-12-07_13-12-39_Wes Size

Control-1	AP(Thermo)1:200+gapdh1:200	14	2	AP	598	66	70969.3	728306.7	43.3	7625.4	9.6	750.8	905.0
Control-2	AP(Thermo)1:200+gapdh1:200	15	1	GAPDH	534	42	99344.3	791577.7	60.7	10000.0	7.5	1421.6	1492.6
Control-2	AP(Thermo)1:200+gapdh1:200	15	2	AP	594	66	47993.1	513405.7	39.3	6485.9	10.0	659.8	1938.4
Control-3	AP(Thermo)1:200+gapdh1:200	16	1	GAPDH	535	41	138039.4	1167037.4	65.9	10000.0	7.9	1795.4	1268.3
Control-3	AP(Thermo)1:200+gapdh1:200	16	2	AP	594	66	57743.1	604477.9	34.1	5179.6	9.8	719.0	1544.7
Control-4	AP(Thermo)1:200+gapdh1:200	17	1	GAPDH	535	41	94694.1	755771.3	60.4	10000.0	7.5	1277.2	1178.4
Control-4	AP(Thermo)1:200+gapdh1:200	17	2	AP	595	66	48015.8	495029.8	39.6	6550.0	9.7	638.8	1409.5
IPS1	AP(Thermo)1:200+gapdh1:200	18	1	GAPDH	535	42	148603.4	1229079.4	65.9	10000.0	7.8	2323.0	1576.2
IPS1	AP(Thermo)1:200+gapdh1:200	18	2	AP	594	66	60677.1	635989.2	34.1	5174.5	9.8	905.4	2042.7

File: 2022-12-07_13-12-39_Wes Size

IPS2	AP(Thermo)1:200 +gapdh1:200	19	1	GAPDH	535	42	168381.8	1559863.0	67.8	10000.0	8.7	1999.3	1678.2
IPS2	AP(Thermo)1:200 +gapdh1:200	19	2	AP	594	66	66377.0	739504.7	32.2	4740.8	10.5	735.2	2162.9
IPS3	AP(Thermo)1:200 +gapdh1:200	20	1	GAPDH	534	41	131499.1	1076995.5	63.6	10000.0	7.7	2201.0	1219.9
IPS3	AP(Thermo)1:200 +gapdh1:200	20	2	AP	593	66	57059.1	616154.4	36.4	5721.0	10.1	917.7	1421.6
IPS3	AP(Thermo)1:200 +gapdh1:200	20	3		647	158	6304.6	178846.9		1660.6	26.6	20.0	1639.0
IPS4	AP(Thermo)1:200 +gapdh1:200	21	1	GAPDH	538	41	183226.7	1741214.1	68.4	10000.0	8.9	2345.8	1303.5
IPS4	AP(Thermo)1:200 +gapdh1:200	21	2	AP	598	66	76531.1	803028.1	31.6	4611.9	9.9	941.9	1497.6
IPS1-2	AP(Thermo)1:200 +gapdh1:200	22	1	GAPDH	535	41	171921.1	1527609.4	62.4	10000.0	8.3	2437.9	1430.0
IPS1-2	AP(Thermo)1:200 +gapdh1:200	22	2	AP	594	65	82114.5	921842.9	37.6	6034.5	10.5	1085.1	1850.4

File: 2022-12-07_13-12-39_Wes Size

IPS2-2	AP(Thermo)1:200+gapdh1:200	23	1	GAPDH	536	41	189236.1	1773405.6	69.1	10000.0	8.8	2302.3	1500.3
IPS2-2	AP(Thermo)1:200+gapdh1:200	23	2	AP	595	65	72324.1	793273.2	30.9	4473.2	10.3	822.1	2037.3
IPS2-2	AP(Thermo)1:200+gapdh1:200	23	3		655	151	6657.9	179288.3		1011.0	25.3	21.0	2609.1
IPS3-2	AP(Thermo)1:200+gapdh1:200	24	1	GAPDH	537	41	152909.1	1263522.5	59.5	10000.0	7.8	1996.9	1552.8
IPS3-2	AP(Thermo)1:200+gapdh1:200	24	2	AP	594	65	76849.2	858548.9	40.5	6794.9	10.5	932.2	2070.9
IPS4-2	AP(Thermo)1:200+gapdh1:200	25	1	GAPDH	544	41	176552.1	1525404.0	66.3	10000.0	8.1	2579.0	1255.2
IPS4-2	AP(Thermo)1:200+gapdh1:200	25	2	AP	600	65	76011.3	773909.7	33.7	5073.5	9.6	1047.9	1465.0

File: 2022-12-07_13-12-39_Wes Size

Standard Plots

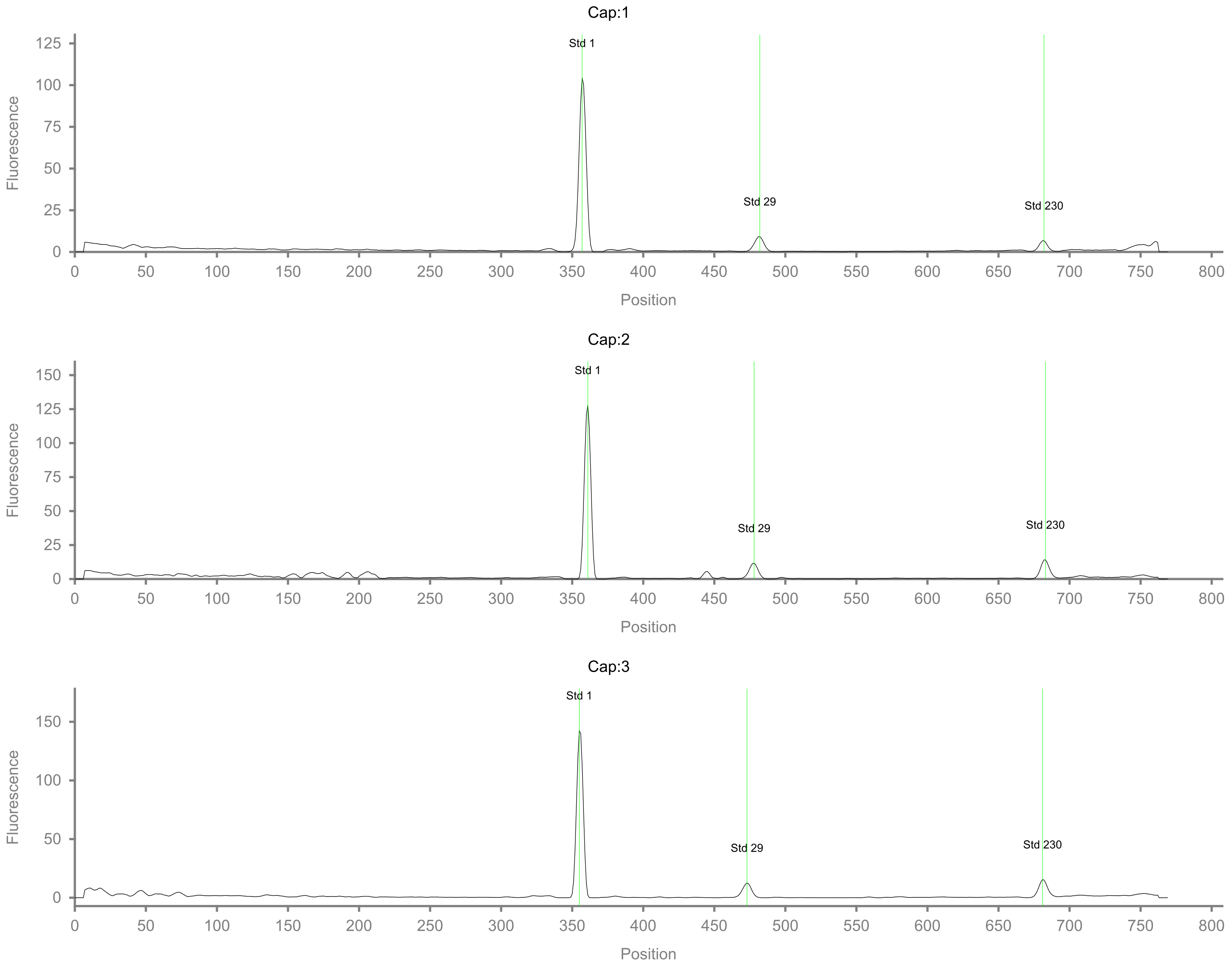

proteinsimple
a biotechne brand

File: 2022-12-07_13-12-39_Wes Size

Cap:4

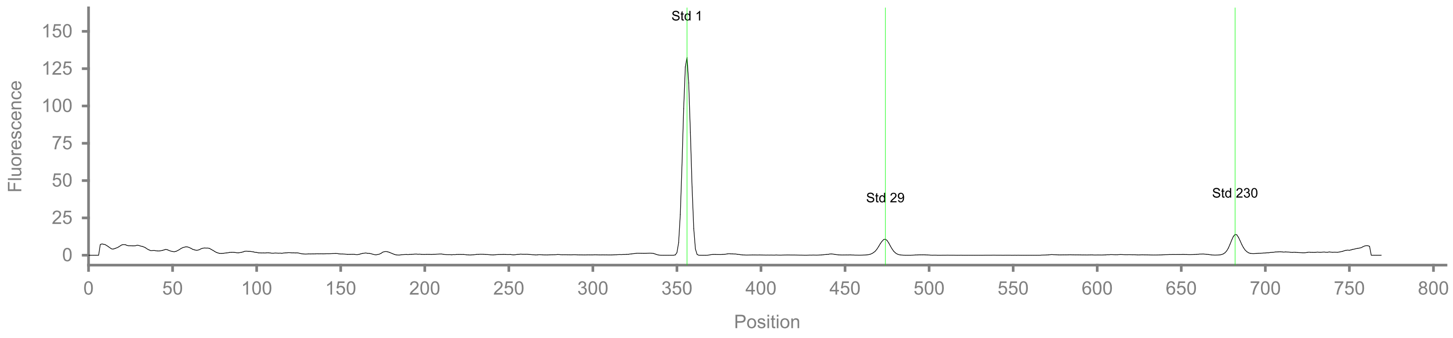

Cap:5

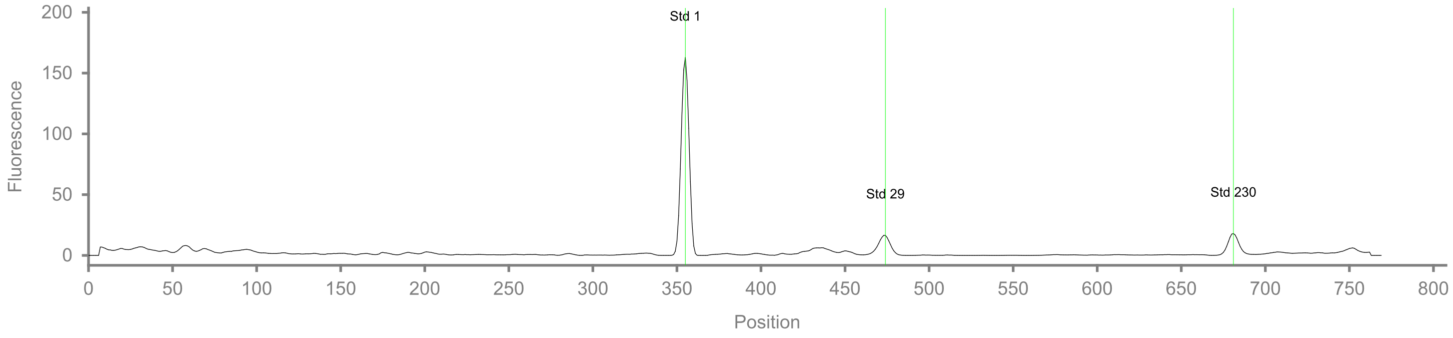

Cap:6

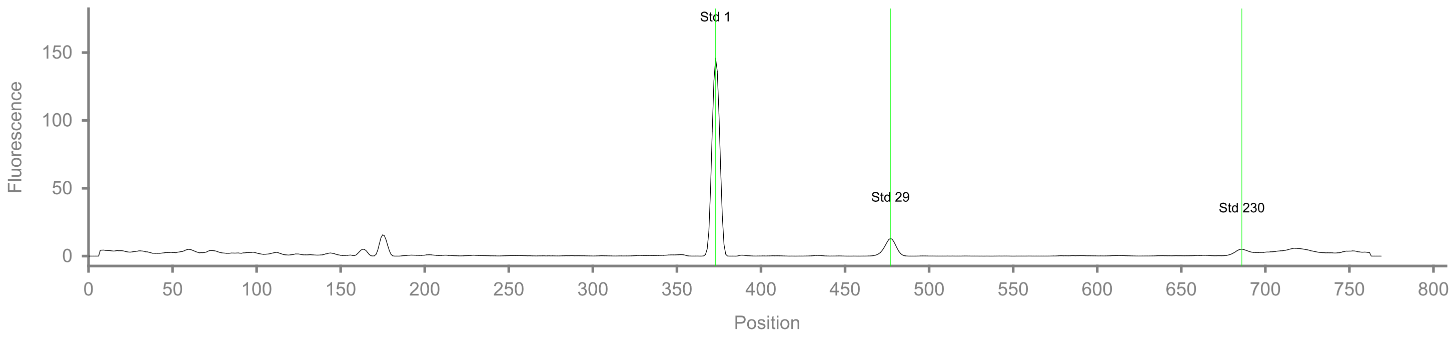

Cap:7

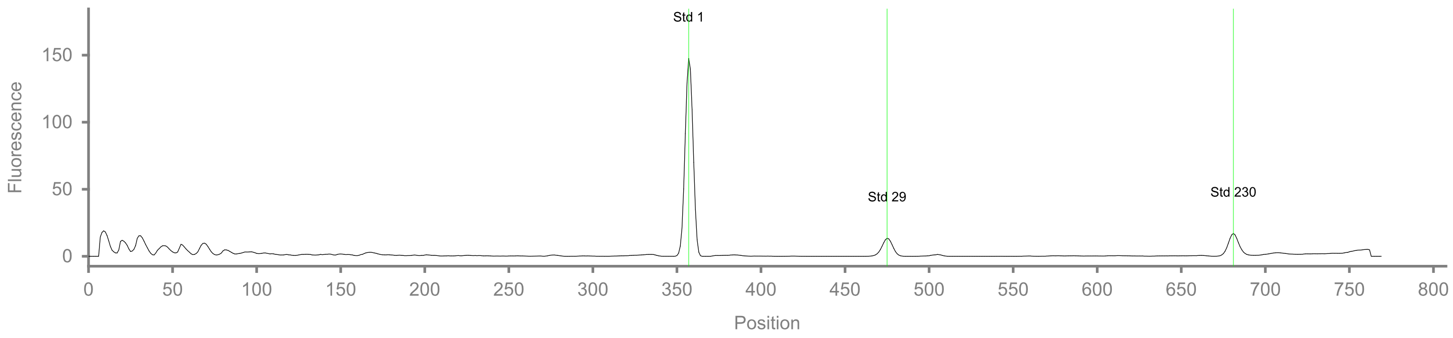

File: 2022-12-07_13-12-39_Wes Size

Cap:8

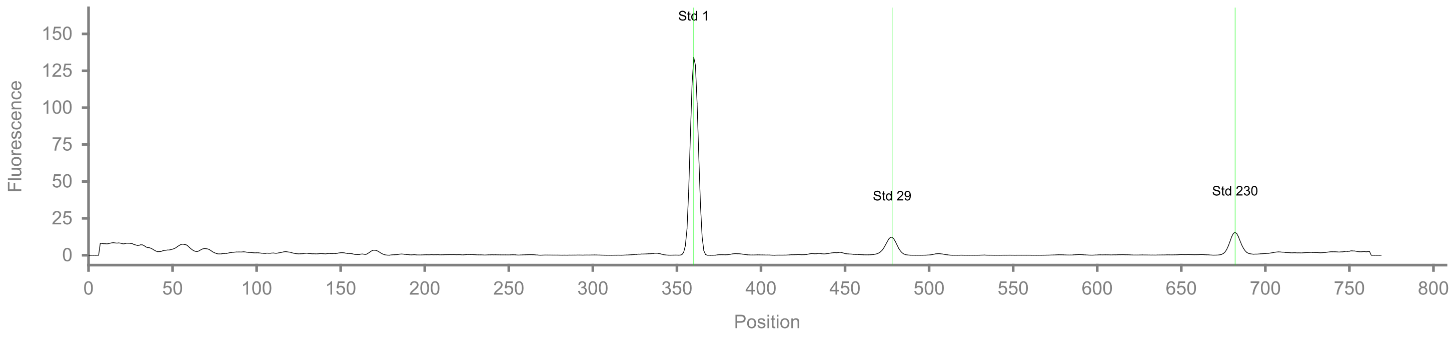

Cap:9

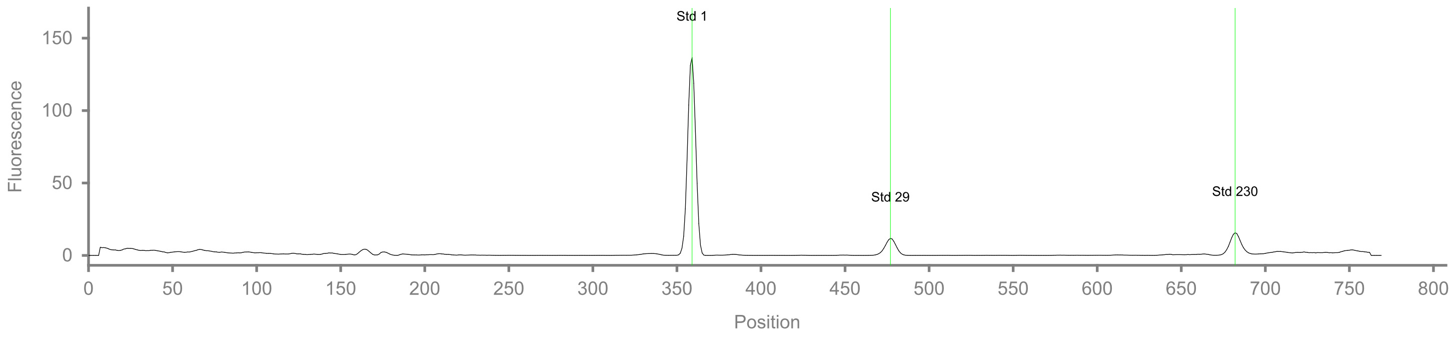

Cap:10

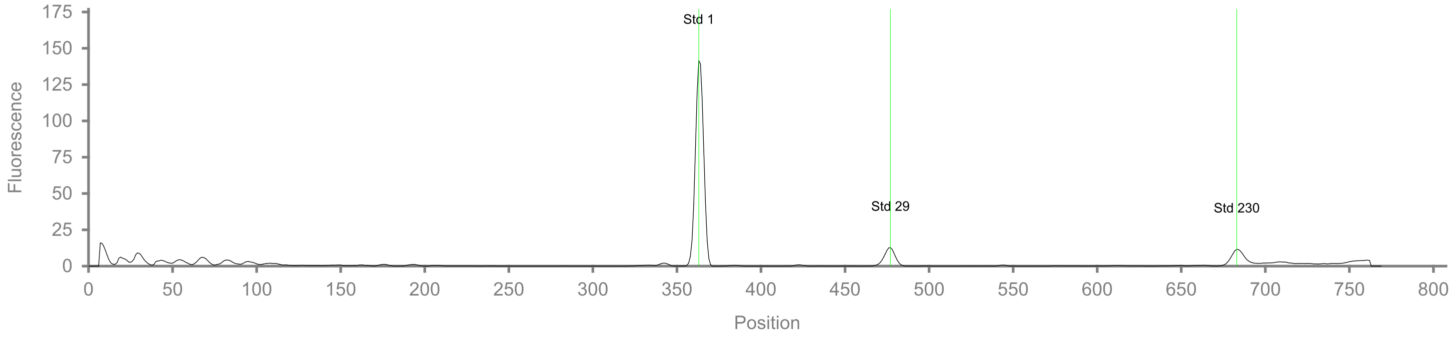

Cap:11

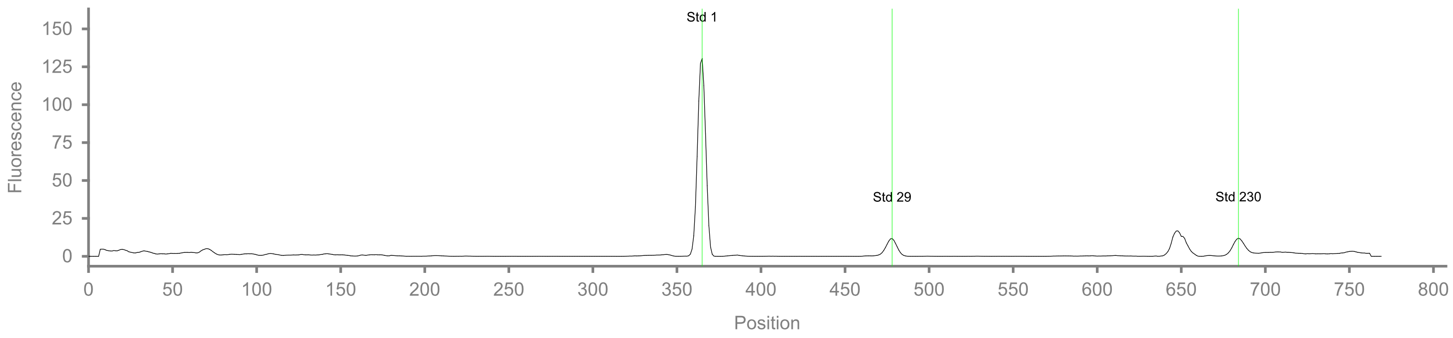

File: 2022-12-07_13-12-39_Wes Size

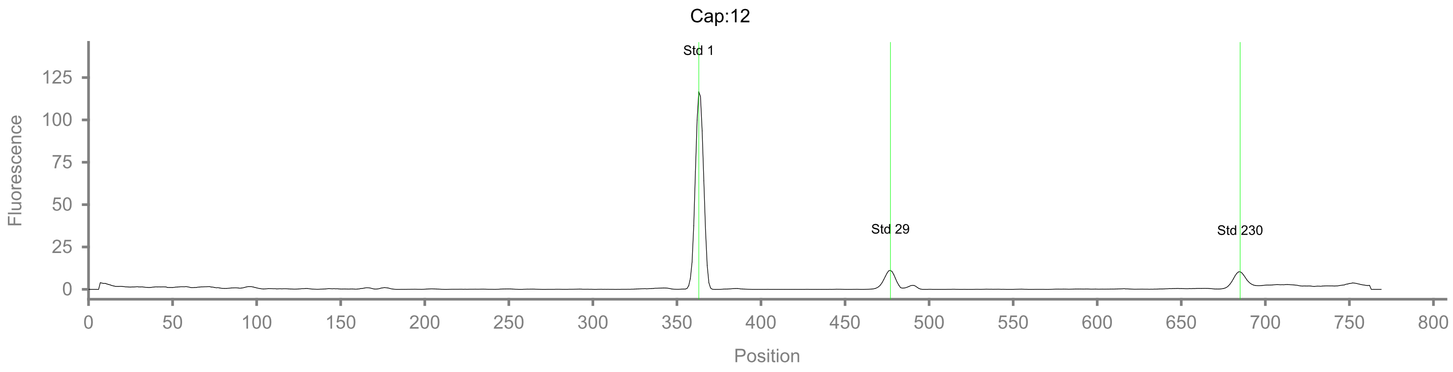

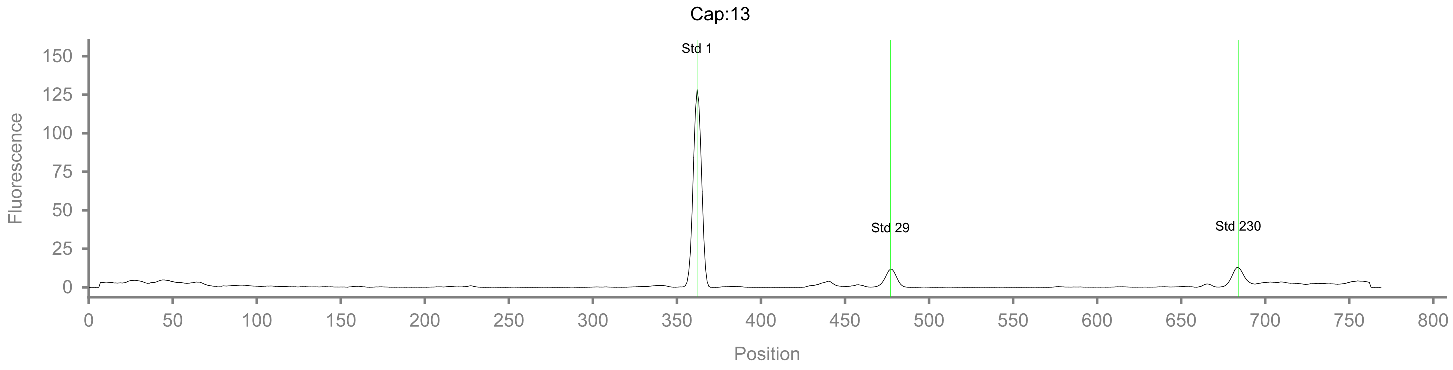

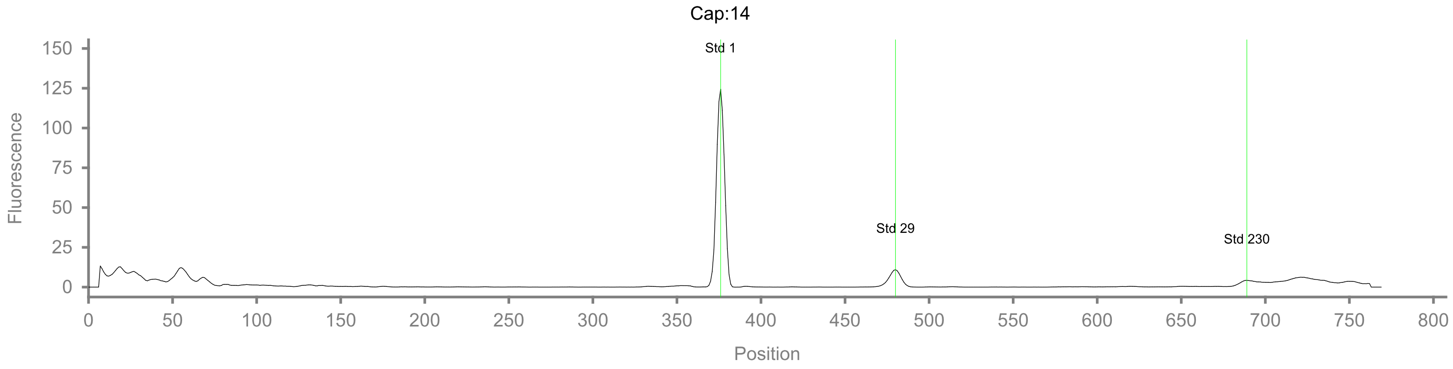

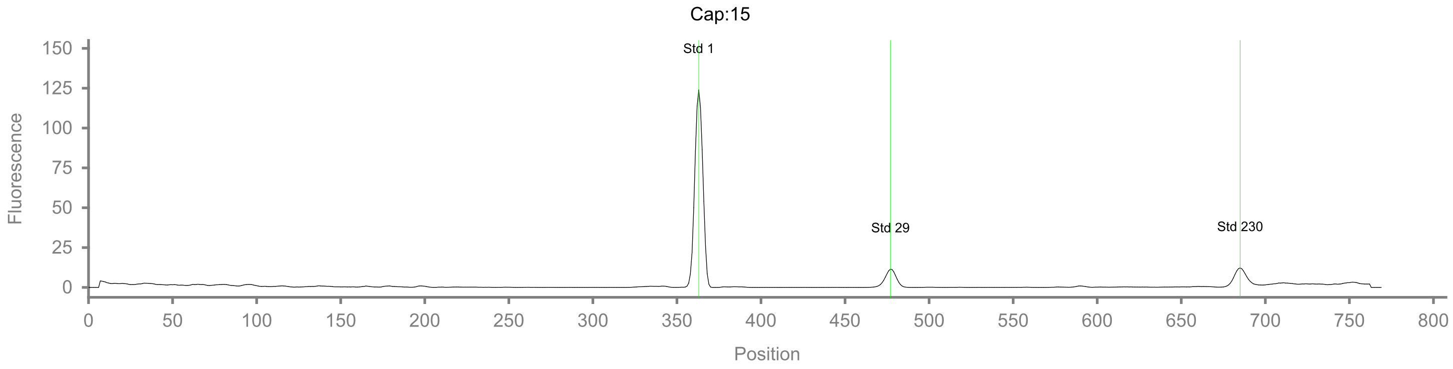

F:\Project\2023 sub\QT342\01 SunW-APCDD1-NC rev8\2022-12-07_13-12-39_Wes Size.cbz

proteinsimple a biotechne brand

File: 2022-12-07_13-12-39_Wes Size

Cap:16

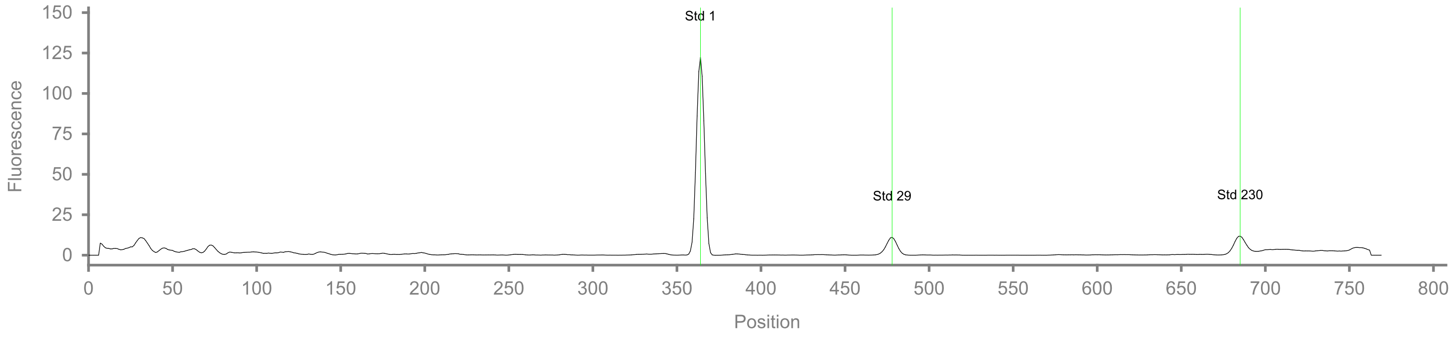

Cap:17

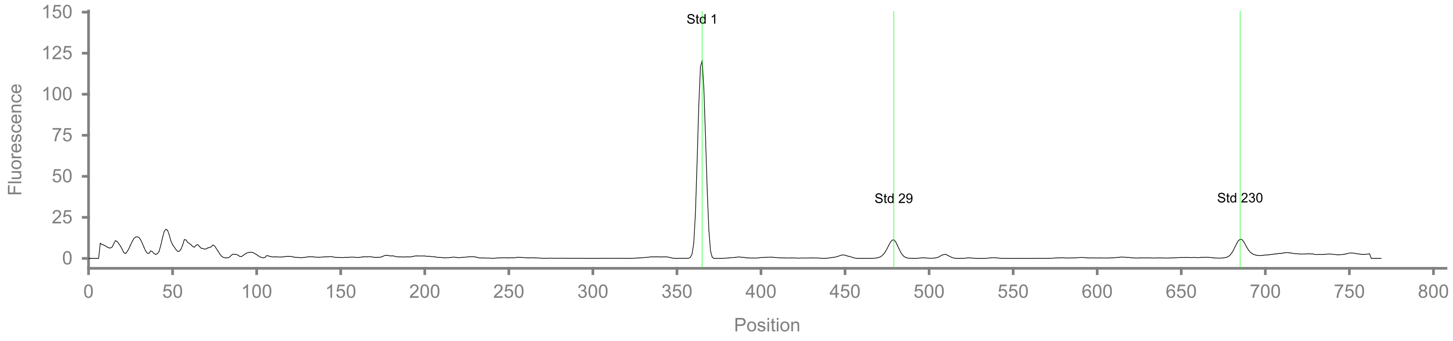

Cap:18

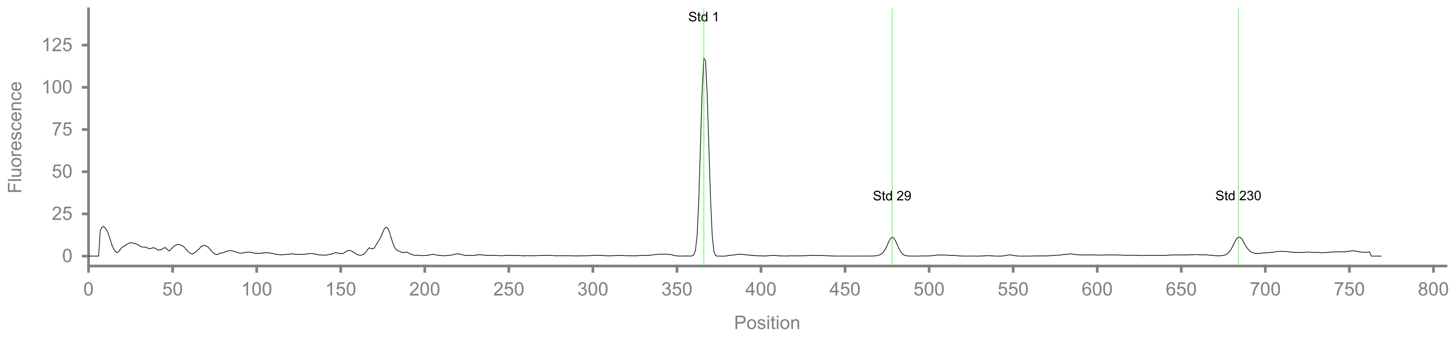

Cap:19

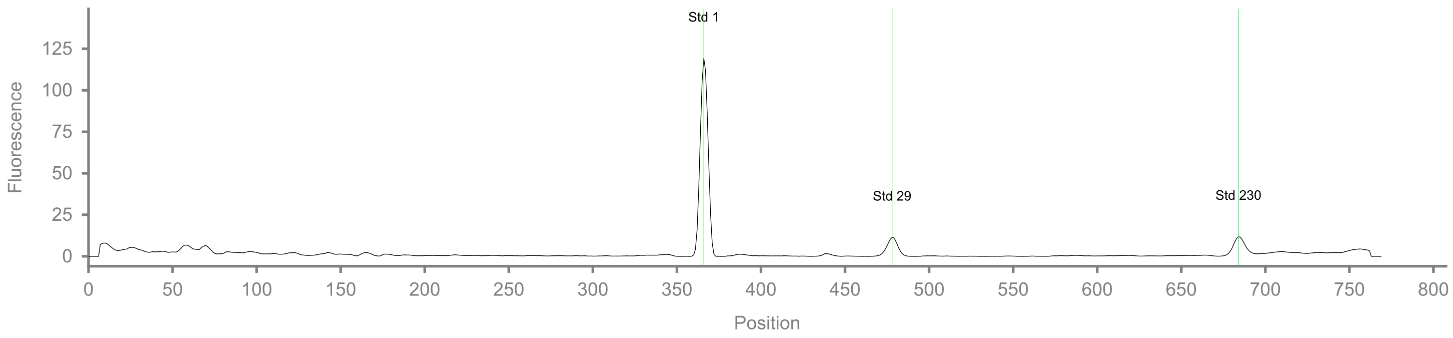

File: 2022-12-07_13-12-39_Wes Size

Cap:20

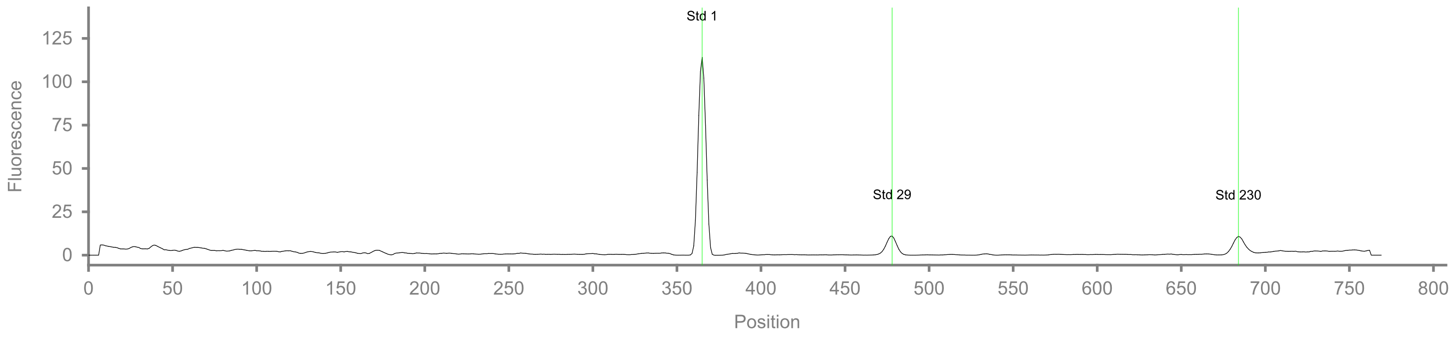

Cap:21

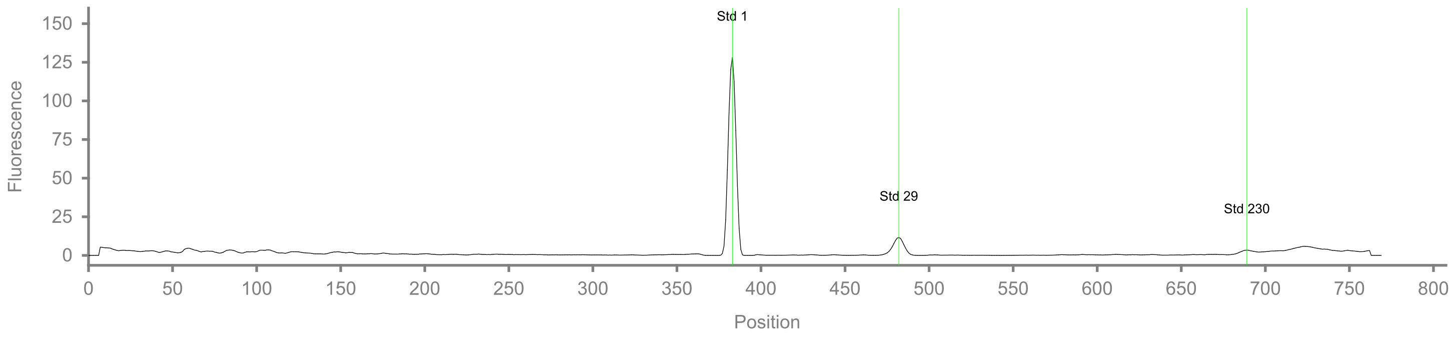

Cap:22

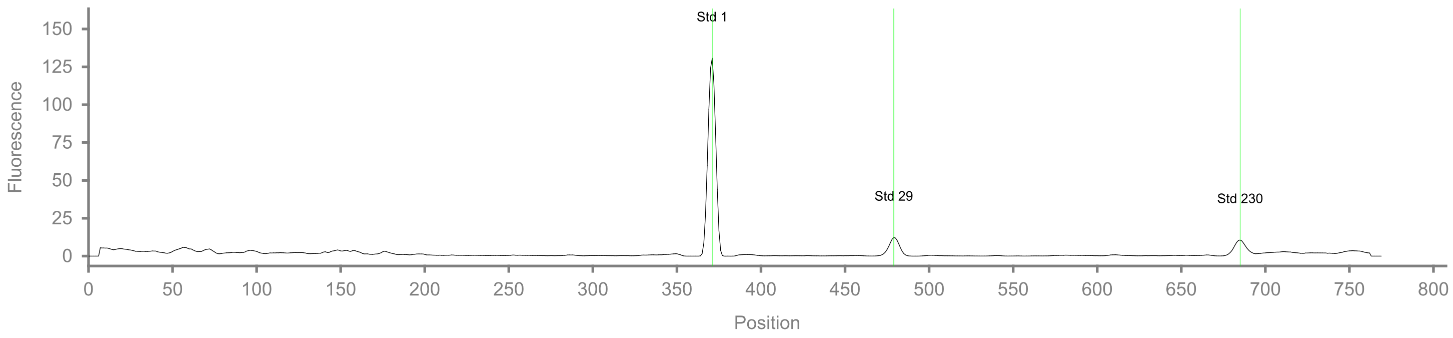

Cap:23

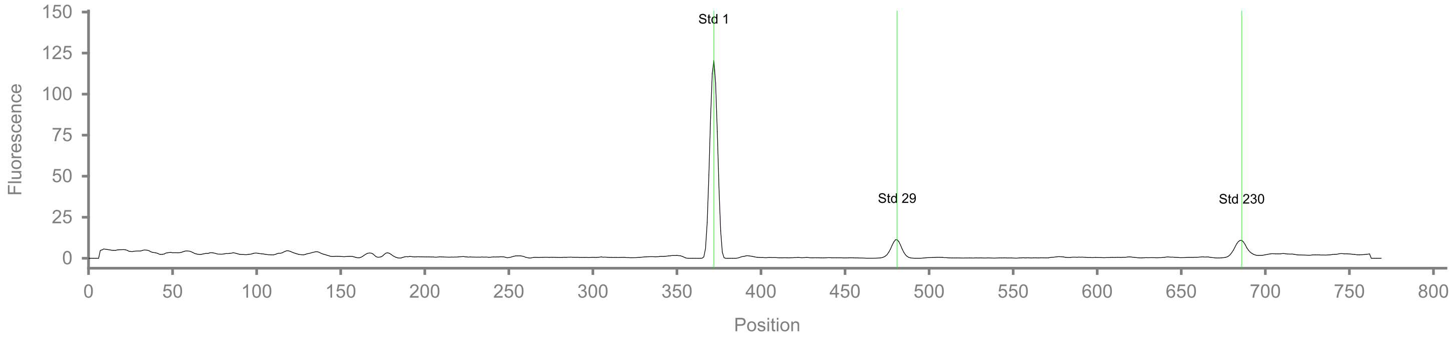

F:\Project\2023 sub\QT342\01 SunW-APCDD1-NC rev8\2022-12-07_13-12-39_Wes Size.cbz

File: 2022-12-07_13-12-39_Wes Size

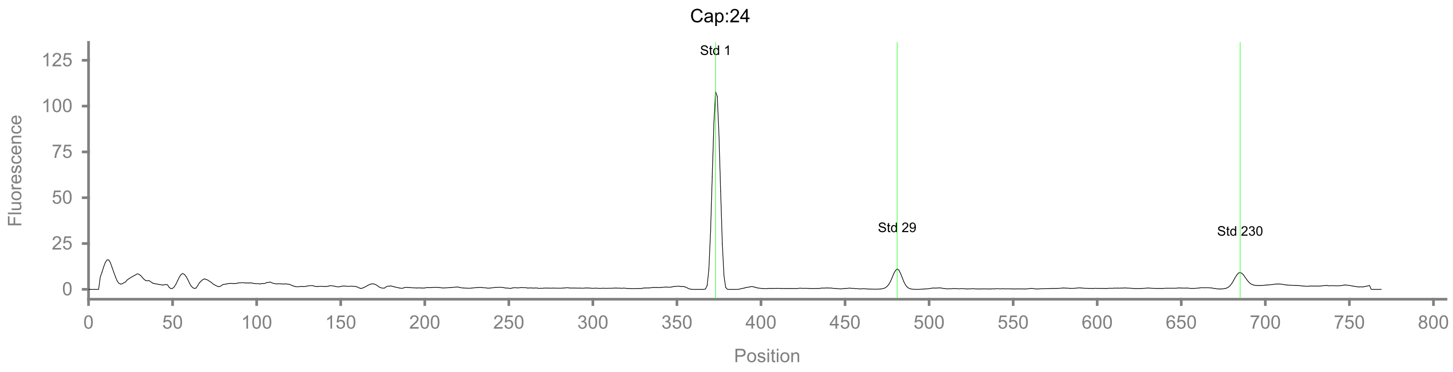

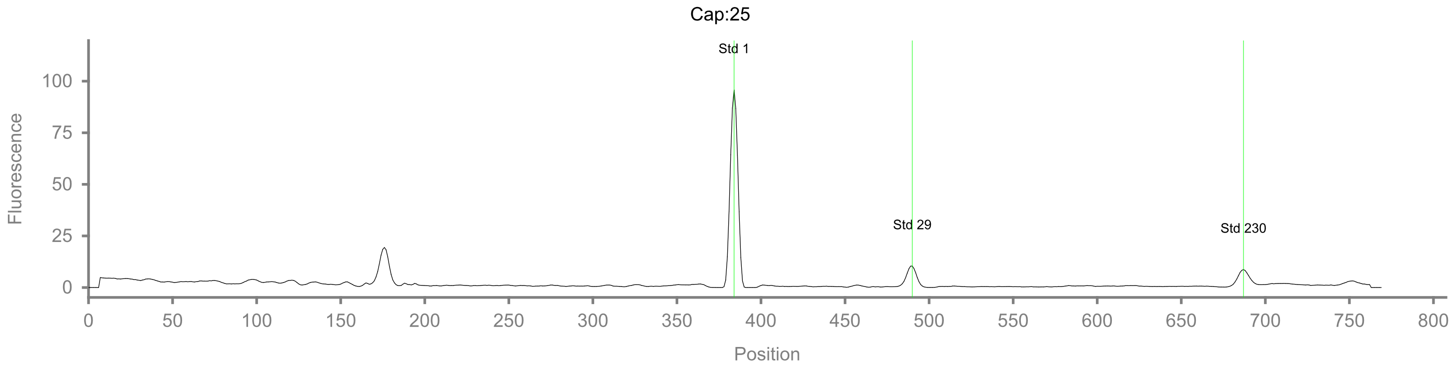

proteinsimple a biotechne brand

File: 2022-12-07_13-12-39_Wes Size

Standard Peaks (pixels)

			Std 1	Std 29	Std 230
Biot. Ladder	Blocking	1	357.0	482.0	682.1
Cell line1	AP(Thermo)1:200+gapdh1:200	2	360.8	477.9	683.2
Cell line2	AP(Thermo)1:200+gapdh1:200	3	355.0	472.9	681.2
Cell line3	AP(Thermo)1:200+gapdh1:200	4	355.7	473.9	682.2
Cell line4	AP(Thermo)1:200+gapdh1:200	5	354.7	473.8	681.2
Cell line5	AP(Thermo)1:200+gapdh1:200	6	372.8	476.9	686.3
Cell line6	AP(Thermo)1:200+gapdh1:200	7	356.8	475.0	681.2
Cell line7	AP(Thermo)1:200+gapdh1:200	8	359.9	477.9	682.2
Cell line 8	AP(Thermo)1:200+gapdh1:200	9	358.6	477.0	682.2
Patient-1	AP(Thermo)1:200+gapdh1:200	10	362.9	476.9	683.3
Patient-2	AP(Thermo)1:200+gapdh1:200	11	364.6	477.9	684.3
Patient-3	AP(Thermo)1:200+gapdh1:200	12	362.9	477.2	685.2
Patient-4	AP(Thermo)1:200+gapdh1:200	13	361.8	477.0	684.2
Control-1	AP(Thermo)1:200+gapdh1:200	14	375.7	479.9	689.3
Control-2	AP(Thermo)1:200+gapdh1:200	15	362.7	477.0	685.2
Control-3	AP(Thermo)1:200+gapdh1:200	16	363.7	477.9	685.3
Control-4	AP(Thermo)1:200+gapdh1:200	17	364.6	478.9	685.2
IPS1	AP(Thermo)1:200+gapdh1:200	18	365.9	478.0	684.2
IPS2	AP(Thermo)1:200+gapdh1:200	19	365.8	478.0	684.2
IPS3	AP(Thermo)1:200+gapdh1:200	20	364.7	477.9	684.2
IPS4	AP(Thermo)1:200+gapdh1:200	21	382.7	481.9	689.2
IPS1-2	AP(Thermo)1:200+gapdh1:200	22	370.7	479.0	685.2
IPS2-2	AP(Thermo)1:200+gapdh1:200	23	371.8	480.9	686.1
IPS3-2	AP(Thermo)1:200+gapdh1:200	24	373.1	481.0	685.2
IPS4-2	AP(Thermo)1:200+gapdh1:200	25	383.9	489.9	687.2

File: 2022-12-07_13-12-39_Wes Size

Grouping

Peak Groups

Cell line 8	AP(Thermo)1:200+gapdh1:200	GAPDH	405448.5			
Cell line 8	AP(Thermo)1:200+gapdh1:200	AP	433704.5			
Cell line1	AP(Thermo)1:200+gapdh1:200	GAPDH	718107.7			
Cell line1	AP(Thermo)1:200+gapdh1:200	AP	710729.4			
Cell line2	AP(Thermo)1:200+gapdh1:200	GAPDH	934328.8			
Cell line2	AP(Thermo)1:200+gapdh1:200	AP	822306.5			
Cell line3	AP(Thermo)1:200+gapdh1:200	GAPDH	407658.3			
Cell line3	AP(Thermo)1:200+gapdh1:200	AP	813779.0			
Cell line4	AP(Thermo)1:200+gapdh1:200	GAPDH	385264.5			
Cell line4	AP(Thermo)1:200+gapdh1:200	AP	606064.2			
Cell line5	AP(Thermo)1:200+gapdh1:200	GAPDH	933655.0			
Cell line5	AP(Thermo)1:200+gapdh1:200	AP	725243.4			
Cell line6	AP(Thermo)1:200+gapdh1:200	GAPDH	691535.4			
Cell line6	AP(Thermo)1:200+gapdh1:200	AP	527941.9			
Cell line7	AP(Thermo)1:200+gapdh1:200	GAPDH	632504.2			
Cell line7	AP(Thermo)1:200+gapdh1:200	AP	526058.2			
Control-1	AP(Thermo)1:200+gapdh1:200	GAPDH	955106.6			
Control-1	AP(Thermo)1:200+gapdh1:200	AP	728306.7			
Control-2	AP(Thermo)1:200+gapdh1:200	GAPDH	791577.7			
Control-2	AP(Thermo)1:200+gapdh1:200	AP	513405.7			
Control-3	AP(Thermo)1:200+gapdh1:200	GAPDH	1167037.4			
Control-3	AP(Thermo)1:200+gapdh1:200	AP	604477.9			

File: 2022-12-07_13-12-39_Wes Size

Control-4	AP(Thermo)1:200+gapdh1:200	GAPDH	755771.3			
Control-4	AP(Thermo)1:200+gapdh1:200	AP	495029.8			
IPS1	AP(Thermo)1:200+gapdh1:200	GAPDH	1229079.4			
IPS1	AP(Thermo)1:200+gapdh1:200	AP	635989.2			
IPS1-2	AP(Thermo)1:200+gapdh1:200	GAPDH	1527609.4			
IPS1-2	AP(Thermo)1:200+gapdh1:200	AP	921842.9			
IPS2	AP(Thermo)1:200+gapdh1:200	GAPDH	1559863.0			
IPS2	AP(Thermo)1:200+gapdh1:200	AP	739504.7			
IPS2-2	AP(Thermo)1:200+gapdh1:200	GAPDH	1773405.6			
IPS2-2	AP(Thermo)1:200+gapdh1:200	AP	793273.2			
IPS3	AP(Thermo)1:200+gapdh1:200	GAPDH	1076995.5			
IPS3	AP(Thermo)1:200+gapdh1:200	AP	616154.4			
IPS3-2	AP(Thermo)1:200+gapdh1:200	GAPDH	1263522.5			
IPS3-2	AP(Thermo)1:200+gapdh1:200	AP	858548.9			
IPS4	AP(Thermo)1:200+gapdh1:200	GAPDH	1741214.1			
IPS4	AP(Thermo)1:200+gapdh1:200	AP	803028.1			
IPS4-2	AP(Thermo)1:200+gapdh1:200	GAPDH	1525404.0			
IPS4-2	AP(Thermo)1:200+gapdh1:200	AP	773909.7			
Patient-1	AP(Thermo)1:200+gapdh1:200	GAPDH	1090377.1			
Patient-1	AP(Thermo)1:200+gapdh1:200	AP	821907.2			
Patient-2	AP(Thermo)1:200+gapdh1:200	GAPDH	846551.3			
Patient-2	AP(Thermo)1:200+gapdh1:200	AP	767851.7			
Patient-3	AP(Thermo)1:200+gapdh1:200	GAPDH	491207.1			
Patient-3	AP(Thermo)1:200+gapdh1:200	AP	779216.4			
Patient-4	AP(Thermo)1:200+gapdh1:200	GAPDH	568345.4			

File: 2022-12-07_13-12-39_Wes Size

Patient-4	AP(Thermo)1:200+gapdh1:200	AP	670406.0			

Cell line 8	AP(Thermo)1:200+gapdh1:200	GAPDH	48.3			
Cell line 8	AP(Thermo)1:200+gapdh1:200	AP	51.7			
Cell line1	AP(Thermo)1:200+gapdh1:200	GAPDH	50.3			
Cell line1	AP(Thermo)1:200+gapdh1:200	AP	49.7			
Cell line2	AP(Thermo)1:200+gapdh1:200	GAPDH	53.2			
Cell line2	AP(Thermo)1:200+gapdh1:200	AP	46.8			
Cell line3	AP(Thermo)1:200+gapdh1:200	GAPDH	33.4			
Cell line3	AP(Thermo)1:200+gapdh1:200	AP	66.6			
Cell line4	AP(Thermo)1:200+gapdh1:200	GAPDH	38.9			
Cell line4	AP(Thermo)1:200+gapdh1:200	AP	61.1			
Cell line5	AP(Thermo)1:200+gapdh1:200	GAPDH	56.3			
Cell line5	AP(Thermo)1:200+gapdh1:200	AP	43.7			
Cell line6	AP(Thermo)1:200+gapdh1:200	GAPDH	56.7			
Cell line6	AP(Thermo)1:200+gapdh1:200	AP	43.3			
Cell line7	AP(Thermo)1:200+gapdh1:200	GAPDH	54.6			
Cell line7	AP(Thermo)1:200+gapdh1:200	AP	45.4			
Control-1	AP(Thermo)1:200+gapdh1:200	GAPDH	56.7			
Control-1	AP(Thermo)1:200+gapdh1:200	AP	43.3			
Control-2	AP(Thermo)1:200+gapdh1:200	GAPDH	60.7			
Control-2	AP(Thermo)1:200+gapdh1:200	AP	39.3			
Control-3	AP(Thermo)1:200+gapdh1:200	GAPDH	65.9			

proteinsimple a biotechne brand

File: 2022-12-07_13-12-39_Wes Size

Control-3	AP(Thermo)1:200+gapdh1:200	AP	34.1			
Control-4	AP(Thermo)1:200+gapdh1:200	GAPDH	60.4			
Control-4	AP(Thermo)1:200+gapdh1:200	AP	39.6			
IPS1	AP(Thermo)1:200+gapdh1:200	GAPDH	65.9			
IPS1	AP(Thermo)1:200+gapdh1:200	AP	34.1			
IPS1-2	AP(Thermo)1:200+gapdh1:200	GAPDH	62.4			
IPS1-2	AP(Thermo)1:200+gapdh1:200	AP	37.6			
IPS2	AP(Thermo)1:200+gapdh1:200	GAPDH	67.8			
IPS2	AP(Thermo)1:200+gapdh1:200	AP	32.2			
IPS2-2	AP(Thermo)1:200+gapdh1:200	GAPDH	69.1			
IPS2-2	AP(Thermo)1:200+gapdh1:200	AP	30.9			
IPS3	AP(Thermo)1:200+gapdh1:200	GAPDH	63.6			
IPS3	AP(Thermo)1:200+gapdh1:200	AP	36.4			
IPS3-2	AP(Thermo)1:200+gapdh1:200	GAPDH	59.5			
IPS3-2	AP(Thermo)1:200+gapdh1:200	AP	40.5			
IPS4	AP(Thermo)1:200+gapdh1:200	GAPDH	68.4			
IPS4	AP(Thermo)1:200+gapdh1:200	AP	31.6			
IPS4-2	AP(Thermo)1:200+gapdh1:200	GAPDH	66.3			
IPS4-2	AP(Thermo)1:200+gapdh1:200	AP	33.7			
Patient-1	AP(Thermo)1:200+gapdh1:200	GAPDH	57.0			
Patient-1	AP(Thermo)1:200+gapdh1:200	AP	43.0			
Patient-2	AP(Thermo)1:200+gapdh1:200	GAPDH	52.4			
Patient-2	AP(Thermo)1:200+gapdh1:200	AP	47.6			
Patient-3	AP(Thermo)1:200+gapdh1:200	GAPDH	38.7			
Patient-3	AP(Thermo)1:200+gapdh1:200	AP	61.3			

File: 2022-12-07_13-12-39_Wes Size

Patient-4	AP(Thermo)1:200+gapdh1:200	GAPDH	45.9			
Patient-4	AP(Thermo)1:200+gapdh1:200	AP	54.1			

Cell line 8	AP(Thermo)1:200+gapdh1:200	GAPDH	10000.0			
Cell line 8	AP(Thermo)1:200+gapdh1:200	AP	10696.9			
Cell line1	AP(Thermo)1:200+gapdh1:200	GAPDH	10000.0			
Cell line1	AP(Thermo)1:200+gapdh1:200	AP	9897.3			
Cell line2	AP(Thermo)1:200+gapdh1:200	GAPDH	10000.0			
Cell line2	AP(Thermo)1:200+gapdh1:200	AP	8801.0			
Cell line3	AP(Thermo)1:200+gapdh1:200	GAPDH	10000.0			
Cell line3	AP(Thermo)1:200+gapdh1:200	AP	19962.3			
Cell line4	AP(Thermo)1:200+gapdh1:200	GAPDH	10000.0			
Cell line4	AP(Thermo)1:200+gapdh1:200	AP	15731.1			
Cell line5	AP(Thermo)1:200+gapdh1:200	GAPDH	10000.0			
Cell line5	AP(Thermo)1:200+gapdh1:200	AP	7767.8			
Cell line6	AP(Thermo)1:200+gapdh1:200	GAPDH	10000.0			
Cell line6	AP(Thermo)1:200+gapdh1:200	AP	7634.3			
Cell line7	AP(Thermo)1:200+gapdh1:200	GAPDH	10000.0			
Cell line7	AP(Thermo)1:200+gapdh1:200	AP	8317.1			
Control-1	AP(Thermo)1:200+gapdh1:200	GAPDH	10000.0			
Control-1	AP(Thermo)1:200+gapdh1:200	AP	7625.4			
Control-2	AP(Thermo)1:200+gapdh1:200	GAPDH	10000.0			
Control-2	AP(Thermo)1:200+gapdh1:200	AP	6485.9			

File: 2022-12-07_13-12-39_Wes Size

Control-3	AP(Thermo)1:200+gapdh1:200	GAPDH	10000.0			
Control-3	AP(Thermo)1:200+gapdh1:200	AP	5179.6			
Control-4	AP(Thermo)1:200+gapdh1:200	GAPDH	10000.0			
Control-4	AP(Thermo)1:200+gapdh1:200	AP	6550.0			
IPS1	AP(Thermo)1:200+gapdh1:200	GAPDH	10000.0			
IPS1	AP(Thermo)1:200+gapdh1:200	AP	5174.5			
IPS1-2	AP(Thermo)1:200+gapdh1:200	GAPDH	10000.0			
IPS1-2	AP(Thermo)1:200+gapdh1:200	AP	6034.5			
IPS2	AP(Thermo)1:200+gapdh1:200	GAPDH	10000.0			
IPS2	AP(Thermo)1:200+gapdh1:200	AP	4740.8			
IPS2-2	AP(Thermo)1:200+gapdh1:200	GAPDH	10000.0			
IPS2-2	AP(Thermo)1:200+gapdh1:200	AP	4473.2			
IPS3	AP(Thermo)1:200+gapdh1:200	GAPDH	10000.0			
IPS3	AP(Thermo)1:200+gapdh1:200	AP	5721.0			
IPS3-2	AP(Thermo)1:200+gapdh1:200	GAPDH	10000.0			
IPS3-2	AP(Thermo)1:200+gapdh1:200	AP	6794.9			
IPS4	AP(Thermo)1:200+gapdh1:200	GAPDH	10000.0			
IPS4	AP(Thermo)1:200+gapdh1:200	AP	4611.9			
IPS4-2	AP(Thermo)1:200+gapdh1:200	GAPDH	10000.0			
IPS4-2	AP(Thermo)1:200+gapdh1:200	AP	5073.5			
Patient-1	AP(Thermo)1:200+gapdh1:200	GAPDH	10000.0			
Patient-1	AP(Thermo)1:200+gapdh1:200	AP	7537.8			
Patient-2	AP(Thermo)1:200+gapdh1:200	GAPDH	10000.0			
Patient-2	AP(Thermo)1:200+gapdh1:200	AP	9070.3			
Patient-3	AP(Thermo)1:200+gapdh1:200	GAPDH	10000.0			

proteinsimple a biotechne brand

File: 2022-12-07_13-12-39_Wes Size

Patient-3	AP(Thermo)1:200+gap dh1:200	AP	15863.3			
Patient-4	AP(Thermo)1:200+gap dh1:200	GAPDH	10000.0			
Patient-4	AP(Thermo)1:200+gap dh1:200	AP	11795.7			

proteinsimple a biotechne brand

Sample Images

Sample, 8s

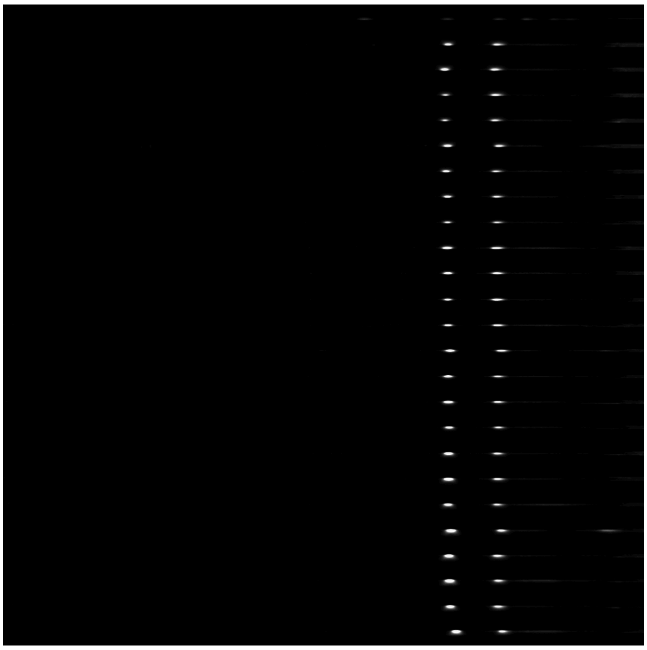

File: 2022-12-07_13-12-39_Wes Size

Standard Images

Std, 4s

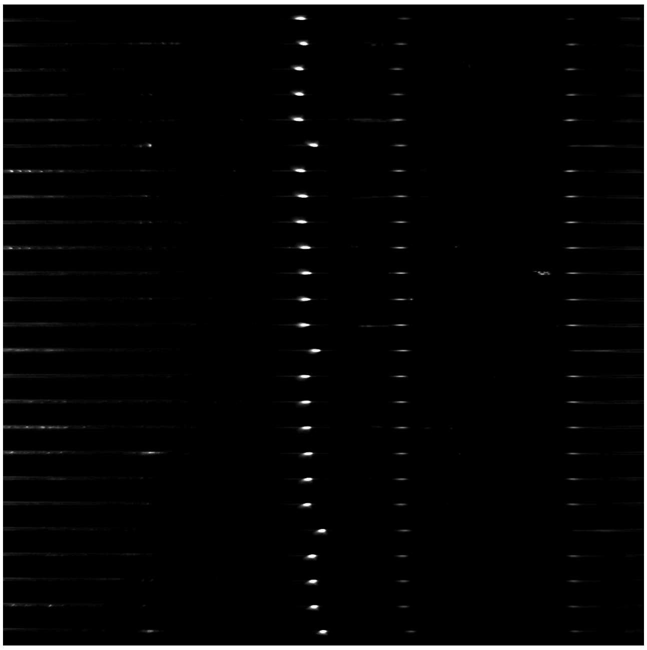

Supplement: Supplementary file 6 — Supplementary Data 3 [file 41467_2024_49376_MOESM6_ESM.pdf]
