# Supplementary material for: Altered chromatin topologies caused by balanced chromosomal translocation lead to central iris hypoplasia
Source: Nat Commun. 2024 Jun 13;15:5048. doi: 10.1038/s41467-024-49376-w (PMC11176186; doi:10.1038/s41467-024-49376-w)
Supplement: Supplementary file 7 — Reporting Summary [file 41467_2024_49376_MOESM7_ESM.pdf]

nature portfolio

Corresponding author(s): Qingjiong Zhang

Last updated by author(s): Apr 23, 2024

Reporting Summary

Nature Portfolio wishes to improve the reproducibility of the work that we publish. This form provides structure for consistency and transparency in reporting. For further information on Nature Portfolio policies, see our Editorial Policies and the Editorial Policy Checklist.

Statistics

For all statistical analyses, confirm that the following items are present in the figure legend, table legend, main text, or Methods section.

n/a	Confirmed	
☐	☒	The exact sample size (*n*) for each experimental group/condition, given as a discrete number and unit of measurement
☐	☒	A statement on whether measurements were taken from distinct samples or whether the same sample was measured repeatedly
☐	☒	The statistical test(s) used AND whether they are one- or two-sided<br>*Only common tests should be described solely by name; describe more complex techniques in the Methods section.*
☒	☐	A description of all covariates tested
☒	☐	A description of any assumptions or corrections, such as tests of normality and adjustment for multiple comparisons
☐	☒	A full description of the statistical parameters including central tendency (e.g. means) or other basic estimates (e.g. regression coefficient) AND variation (e.g. standard deviation) or associated estimates of uncertainty (e.g. confidence intervals)
☐	☒	For null hypothesis testing, the test statistic (e.g. *F*, *t*, *r*) with confidence intervals, effect sizes, degrees of freedom and *P* value noted<br>*Give P values as exact values whenever suitable.*
☒	☐	For Bayesian analysis, information on the choice of priors and Markov chain Monte Carlo settings
☒	☐	For hierarchical and complex designs, identification of the appropriate level for tests and full reporting of outcomes
☒	☐	Estimates of effect sizes (e.g. Cohen's *d*, Pearson's *r*), indicating how they were calculated

Our web collection on statistics for biologists contains articles on many of the points above.

Software and code

Policy information about availability of computer code

Data collection	HiC libraries were sequenced on the Illumina NovaSeq platform in PE150 mode. RNA-seq libraries were sequenced on the Illumina HiSeq platform in PE150 mode. CUT&Tag libraries were sequenced on illumina Nova 6000 platform. qPCR was performed employing QuantStudio Dx (ThermoFisher, MA, USA). Simple Western analysis was performed using the ProteinSimple Wes Simple Western system with a 12-230 kDa Master Kit (Proteinsimple, Santa Clara, CA). The whole body and ocular at lateral side of zebrafish larvae were captured by stereoscopic fluorescence microscope M205FA (Leica, Germany). Images of immunofluorescence staining were captured using a Zeiss LSM980 (Carl Zeiss, Baden-wurberg, Germany) confocal microscope.
Data analysis	For RNA-seq, reads were aligned to human reference genome (hg38) using hisat2 (v2.1.0). Low-quality mapping reads were filtered using samtools (v1.3.1). The coverage of sorted reads across the genome was calculated using deepTools (v3.4.3) with the parameter '--binsize 10'. Reads of each gene using 'htseq-count' function of HTSeq (v0.11.2). Differential expressed analysis was performed using DEseq2 (1.30.1) R package, with thresholds '|log2(fold change)|>1 and FDR<0.05'. Volcano plot of differentially expressed genes were generated using custom R code and ggplot2 (v3.3.3) package.<br>For CUT&Tag, reads were aligned to the human reference genome (hg38) using BWA (v0.7.15) with default parameters. Low-quality mapping reads were filtered using samtools (v1.3.1). PCR duplicates were removed using Picard (v 1.107). The coverage of uniquely mapped reads coverage was calculated using 'bamCoverage' function of deepTools (v3.4.3) with the parameter '--binsize 10'. H3K27ac modification peaks were identified using MACS2 (v2.2.7.1) with default parameters.<br>For Hi-C, reads were processed using HiC-Pro pipline (v2.11.1). The contact maps were subjected to normalization using iterative correction and eigenvector decomposition method. The normalized contact maps were converted to h5 format using HiCExplorer (v3.5.1). The contact maps were visualized using HiCExplorer (v3.5.1).<br>For long-read whole-genome sequencing, reads were aligned with human reference genome (hg38) using NGMLR (v0.2.7) and alignments

were visualized in IGV.
Code availability. We have made use of publicly available software and tools.

For manuscripts utilizing custom algorithms or software that are central to the research but not yet described in published literature, software must be made available to editors and reviewers. We strongly encourage code deposition in a community repository (e.g. GitHub). See the Nature Portfolio guidelines for submitting code & software for further information.

Data

Policy information about availability of data

All manuscripts must include a data availability statement. This statement should provide the following information, where applicable:

- Accession codes, unique identifiers, or web links for publicly available datasets
- A description of any restrictions on data availability
- For clinical datasets or third party data, please ensure that the statement adheres to our policy

The data from Long-read WGS, Hi-C, RNA-Seq, and CUT&Tag reported here were deposited in the Genome Sequence Archive (GSA) database under accession code HRA004690 and can be publicly accessible at https://ngdc.cncb.ac.cn/gsa-human/browse/HRA004690. Source data are provided with this paper.

Research involving human participants, their data, or biological material

Policy information about studies with human participants or human data. See also policy information about sex, gender (identity/presentation), and sexual orientation and race, ethnicity and racism.

Reporting on sex and gender	Sex and/or gender of participants was not considered in the study design either in recruitment of patients with central iris hypoplasia or in the investigation of genetic basis of the disease. Sex and/or gender of participants was determined based on self-report
Reporting on race, ethnicity, or other socially relevant groupings	No socially relevant categorization variables were used in our manuscript. Five probands with central iris hypoplasia involving pupillary zone and their available family members, including a four-generation family (#71342), were identified from Zhongshan Ophthalmic Center, Guangzhou, China. Written informed consent in accordance with the tenets of the Declaration of Helsinki was obtained from all participants or their guardians prior to this study. This study was approved by the Institutional Review Board of the Zhongshan Ophthalmic Center, Sun Yat-sen University (2011KYNL012).
Population characteristics	Cases with central iris hypoplasia affecting the pupillary zone with an exam age range from one year old to 72 years old. Aside from genetic investigation, the cases underwent routine ophthalmic examinations, including best-corrected visual acuity, slit lamp biomicroscope, direct ophthalmoscopy, fundus photography, and optical coherence tomography.
Recruitment	Patients with central iris hypoplasia without abnormalities of cornea, lens, retina or fovea were recruited in this study. Clinical data and peripheral blood were collected from probands with central iris hypoplasia and their available family members. Written informed consent in accordance with the tenets of the Declaration of Helsinki was obtained from all participants or their guardians prior to this study.
Ethics oversight	Clinical data and peripheral blood were collected from probands with central iris hypoplasia and their available family members under approval by the Institutional Review Board of the Zhongshan Ophthalmic Center, Sun Yat-sen University (2011KYNL012). The generation of iPSCs from peripheral blood of participants received ethics approval from the Institutional Review Board of the Zhongshan Ophthalmic Center, Sun Yat-sen University (2011KYNL012). The use of postmortem human ocular tissues, which were obtained from the Eye Bank of Guangdong Province, was approved by the Institutional Review Board of the Zhongshan Ophthalmic Center, Sun Yat-sen University (2023KYPJ200).

Note that full information on the approval of the study protocol must also be provided in the manuscript.

Field-specific reporting

Please select the one below that is the best fit for your research. If you are not sure, read the appropriate sections before making your selection.

☒ Life sciences ☐ Behavioural & social sciences ☐ Ecological, evolutionary & environmental sciences

For a reference copy of the document with all sections, see nature.com/documents/nr-reporting-summary-flat.pdf

Life sciences study design

All studies must disclose on these points even when the disclosure is negative.

Sample size	No sample-size calculation was performed for the extremely rare phenotype descried in this study. Five probands, including one from a large family with 12 family members, of which 6 were affected, were recruited in this study.
Data exclusions	No data were excluded from the analyses.
Replication	Sanger sequencing was used to validate the breakpoints of the translocation and the results showed that all the six affected members had the same translocation variation. The number of zebrafish tested was 782 and RT-qPCR was carried out in quadruplicate. All attempts at replication were successful.

Randomization	Randomization was not applicable to the study because the inclusion criteria of these participants were all have the same phenotype.
Blinding	Blinding was not possible because the molecular basis of the disease was explored based on a definite phenotypic information of participants.

Behavioural & social sciences study design

All studies must disclose on these points even when the disclosure is negative.

Study description	N/A
Research sample	N/A
Sampling strategy	*Describe the sampling procedure (e.g. random, snowball, stratified, convenience). Describe the statistical methods that were used to predetermine sample size OR if no sample-size calculation was performed, describe how sample sizes were chosen and provide a rationale for why these sample sizes are sufficient. For qualitative data, please indicate whether data saturation was considered, and what criteria were used to decide that no further sampling was needed.*
Data collection	N/A
Timing	N/A
Data exclusions	N/A
Non-participation	N/A
Randomization	N/A

Ecological, evolutionary & environmental sciences study design

All studies must disclose on these points even when the disclosure is negative.

Study description	N/A
Research sample	N/A
Sampling strategy	N/A
Data collection	N/A
Timing and spatial scale	N/A
Data exclusions	N/A
Reproducibility	N/A
Randomization	N/A
Blinding	N/A

Did the study involve field work? ☐ Yes ☐ No

Field work, collection and transport

Field conditions	N/A
Location	N/A
Access & import/export	N/A
Disturbance	N/A

Reporting for specific materials, systems and methods

We require information from authors about some types of materials, experimental systems and methods used in many studies. Here, indicate whether each material, system or method listed is relevant to your study. If you are not sure if a list item applies to your research, read the appropriate section before selecting a response.

Materials & experimental systems

n/a	Involved in the study
☐	☒ Antibodies
☐	☒ Eukaryotic cell lines
☒	☐ Palaeontology and archaeology
☐	☒ Animals and other organisms
☒	☐ Clinical data
☒	☐ Dual use research of concern
☒	☐ Plants

Methods

n/a	Involved in the study
☒	☐ ChIP-seq
☒	☐ Flow cytometry
☒	☐ MRI-based neuroimaging

Antibodies

Antibodies used

Anti-APCDD1 (Bioss, Bs-1565R), APCDD1 Polyclonal Antibody (Thermo Fisher Scientific, PA5-98605), GAPDH (14C10) Rabbit mAb (Cell Signaling Technology, 2118S), Podoplanin Monoclonal Antibody (Thermo Fisher Scientific, 14-5381-82), Anti-Actin, α-Smooth Muscle - FITC antibody (Sigma-Aldrich, F3777), Mouse CDO Antibody (R&D systems, AF2429-SP), Anti-β-Tubulin III antibody (Sigma-Aldrich, T8578), Histone H3K27ac antibody (Active motif, 39133), Anti-Rabbit IgG (H+L) (Sigma-Aldrich, SAB3700894), Donkey Anti-Mouse IgG H&L (Alexa Fluor® 488) (Abcam, ab150105), and Donkey Anti-Rabbit IgG H&L (Alexa Fluor® 568) (Abcam, ab175470).

Validation

Anti-APCDD1 (#PA5-98605, 1:200 dilution, Thermo Fisher Scientific) and anti-GAPDH (#2118S, clone 14C10, 1:200 dilution, Cell Signaling Technology) were used for Simple Western analysis in induced pluripotent stem cells from the patient and control. Anti-APCDD1 (#Bs-1565R, 1:200 dilution, Bioss), anti-Podoplanin (#14-5381-82, clone eBio8.1.1 (8.1.1), 1:200 dilution, Thermo Fisher Scientific), anti-α-SMA (#F3777, clone 1A4, 1:500 dilution, Sigma-Aldrich), Donkey Anti-Mouse IgG H&L (Alexa Fluor® 488) (#ab150105, 1:1000 dilution, Abcam), and Donkey Anti-Rabbit IgG H&L (Alexa Fluor® 568) (#ab175470, 1:1000 dilution, Abcam) were used for immunofluorescence staining in human eyes. Anti-APCDD1 (#Bs-1565R, 1:200 dilution, Bioss), anti-CDO (#AF2429-SP, 1:200 dilution, R&D systems), Anti-β-Tubulin III (#T8578, clone 2G10, 1:500 dilution, Sigma-Aldrich), Donkey Anti-Mouse IgG H&L (Alexa Fluor® 488) (#ab150105, 1:1000 dilution, Abcam), and Donkey Anti-Rabbit IgG H&L (Alexa Fluor® 568) (#ab175470, 1:1000 dilution, Abcam) were used for immunofluorescence staining in mouse eyes. Histone H3K27ac (#39133, 1:50 dilution, Active motif) and anti-Rabbit IgG (H+L) (#SAB3700894, 1:50 dilution, Sigma-Aldrich) was used for CUT&Tag in human iPSC. All validation statements of antibodies were provided in the Methods section of the manuscript and Supplementary Table 5.

Eukaryotic cell lines

Policy information about cell lines and Sex and Gender in Research

Cell line source(s)

Induced pluripotent stem cells (iPSCs) from a patient and a control.

Authentication

Heterozygous translocation was identified in the iPSCs from the patient, whereas the translocation was not identified in the iPSCs from the control.

Mycoplasma contamination

The two iPSCs were tested negative for mycoplasma contamination.

Commonly misidentified lines
(See ICLAC register)

N/A

Palaeontology and Archaeology

Specimen provenance

N/A

Specimen deposition

N/A

Dating methods

N/A

☐ Tick this box to confirm that the raw and calibrated dates are available in the paper or in Supplementary Information.

Ethics oversight

N/A

Note that full information on the approval of the study protocol must also be provided in the manuscript.

Animals and other research organisms

Policy information about studies involving animals; ARRIVE guidelines recommended for reporting animal research, and Sex and Gender in Research

Laboratory animals	Mice used in this study have a congenic C57BL/6J background and were derived by backcrossing to a parental inbred strain for at least ten generations. They were bred and housed in a specific pathogen-free mouse facility with a regular 12 h light and 12 h dark cycle and persistent environment temperature ranging between 20 °C and 22 °C with 40-60% humidity. Eyes from wild-type mice (C57BL/6J) with different developmental stages, including embryonic stages E11.5, E13.5, E14.5, E17.5, and postnatal stages P1, P14, 1M, 2M, were used for immunofluorescence experiment. Wild-type zebrafish (AB Danio rerio) embryos were acquired from the Zebrafish Facility, Laboratory Animal Center, Sun Yat-sen University. The embryos were bred by natural spawning and maintained at 28.5°C in a constant temperature with a 14 h light: 10 h dark cycle. Zebrafish embryos and larvae from wild-type AB strains were used for microinjections and immunofluorescence experiments.
Wild animals	No wild animals were used in this study.
Reporting on sex	Sex was not considered in study design because it would not cause protein staining differences in immunofluorescence experiments. Sex was not considered in study design, as it is not determined at the early developmental stages studied in this study.
Field-collected samples	This study did not involve samples collected from the field.
Ethics oversight	All animal experiments were performed according to the Association for Research in Vision and Ophthalmology (ARVO) Statement for the Use of Animals in Ophthalmic and Vision Research and guidelines established by the Animal Experimental Ethics Committee of Zhongshan Ophthalmic Center, Sun Yat-sen University (W2021005-1 for mouse experiments and 2016-196 for zebrafish experiments).

Note that full information on the approval of the study protocol must also be provided in the manuscript.

Dual use research of concern

Policy information about dual use research of concern

Hazards

Could the accidental, deliberate or reckless misuse of agents or technologies generated in the work, or the application of information presented in the manuscript, pose a threat to:

No	Yes	
☒	☐	Public health
☒	☐	National security
☒	☐	Crops and/or livestock
☒	☐	Ecosystems
☒	☐	Any other significant area

Experiments of concern

Does the work involve any of these experiments of concern:

No	Yes	
☒	☐	Demonstrate how to render a vaccine ineffective
☒	☐	Confer resistance to therapeutically useful antibiotics or antiviral agents
☒	☐	Enhance the virulence of a pathogen or render a nonpathogen virulent
☒	☐	Increase transmissibility of a pathogen
☒	☐	Alter the host range of a pathogen
☒	☐	Enable evasion of diagnostic/detection modalities
☒	☐	Enable the weaponization of a biological agent or toxin
☒	☐	Any other potentially harmful combination of experiments and agents

Plants

Seed stocks	N/A
Novel plant genotypes	N/A

Authentication | N/A

ChIP-seq

Data deposition

- [ ] Confirm that both raw and final processed data have been deposited in a public database such as GEO.
- [ ] Confirm that you have deposited or provided access to graph files (e.g. BED files) for the called peaks.

Data access links<br>*May remain private before publication.*	N/A
Files in database submission	N/A
Genome browser session<br>(e.g. UCSC)	N/A

Methodology

Replicates	N/A
Sequencing depth	N/A
Antibodies	N/A
Peak calling parameters	N/A
Data quality	N/A
Software	N/A

Flow Cytometry

Plots

Confirm that:

- [ ] The axis labels state the marker and fluorochrome used (e.g. CD4-FITC).
- [ ] The axis scales are clearly visible. Include numbers along axes only for bottom left plot of group (a 'group' is an analysis of identical markers).
- [ ] All plots are contour plots with outliers or pseudocolor plots.
- [ ] A numerical value for number of cells or percentage (with statistics) is provided.

Methodology

Sample preparation	N/A
Instrument	N/A
Software	N/A
Cell population abundance	N/A
Gating strategy	N/A

- [ ] Tick this box to confirm that a figure exemplifying the gating strategy is provided in the Supplementary Information.

Magnetic resonance imaging

Experimental design

Design type	N/A
Design specifications	N/A
Behavioral performance measures	N/A

Acquisition

Imaging type(s)	N/A
Field strength	N/A
Sequence & imaging parameters	N/A
Area of acquisition	N/A

Diffusion MRI ☐ Used ☐ Not used

Preprocessing

Preprocessing software	N/A
Normalization	N/A
Normalization template	N/A
Noise and artifact removal	N/A
Volume censoring	N/A

Statistical modeling & inference

Model type and settings	N/A
Effect(s) tested	N/A

Specify type of analysis: ☐ Whole brain ☐ ROI-based ☐ Both

Statistic type for inference (See Eklund et al. 2016)	N/A
Correction	N/A

Models & analysis

n/a	Involved in the study
☐	☐ Functional and/or effective connectivity
☐	☐ Graph analysis
☐	☐ Multivariate modeling or predictive analysis

Functional and/or effective connectivity	N/A
Graph analysis	N/A
Multivariate modeling and predictive analysis	N/A
